# Comparative Genomic Analyses of Lactococcus garvieae Isolated from Bovine Mastitis in China

**DOI:** 10.1128/spectrum.02995-22

**Published:** 2023-05-08

**Authors:** Yushan Lin, Jinge Han, Herman W. Barkema, Yue Wang, Jian Gao, John P. Kastelic, Bo Han, Shunyi Qin, Zhaoju Deng

**Affiliations:** a Department of Clinical Veterinary Medicine, College of Veterinary Medicine, China Agricultural University, Beijing, People’s Republic of China; b College of Animal Science and Veterinary Medicine, Tianjin Agricultural University, Tianjin, People’s Republic of China; c Faculty of Veterinary Medicine, University of Calgary, Calgary, Alberta, Canada; Shanghai Veterinary Research Institute, Chinese Academy of Sciences

**Keywords:** bovine mastitis, *Lactococcus garvieae*, population structure, virulence genes, antimicrobial resistance, host adaptation

## Abstract

Lactococcus garvieae is an emerging zoonotic pathogen, but there are few reports regarding bovine mastitis. The prevalence of L. garvieae represents an increasing disease threat and global public health risk. Thirty-nine L. garvieae isolates were obtained from 2,899 bovine clinical mastitis milk samples in 6 provinces of China from 2017 to 2021. Five clonal complexes were determined from 32 multilocus sequence types (MLSTs) of L. garvieae: sequence type 46 (ST46) was the predominant sequence type, and 13 novel MLSTs were identified. All isolates were resistant to chloramphenicol and clindamycin, but susceptible to penicillin, ampicillin, amoxicillin-clavulanic acid, imipenem, ceftiofur, enrofloxacin, and marbofloxacin. Based on genomic analyses, L. garvieae had 6,310 genes, including 1,015 core, 3,641 accessory, and 1,654 unique genes. All isolates had virulence genes coding for collagenase, fibronectin-binding protein, glyceraldehyde-3-phosphate dehydrogenase, superoxide dismutase, and NADH oxidase. Most isolates had *lsaD* and *mdtA* antimicrobial resistance (AMR) genes. Based on COG (Clusters of Orthologous Genes database) results, the functions of defense, transcription and replication, and recombination and repair were enhanced in unique genes, whereas functions of translation, ribosomal structure, and biogenesis were enhanced in core genes. The KEGG functional categories enriched in unique genes included human disease and membrane transport, whereas COG functional categories enriched in core genes included energy metabolism, nucleotide metabolism, and translation. No gene was significantly associated with host specificity. In addition, analysis of core genome single nucleotide polymorphisms (SNPs) implied potential host adaptation of some isolates in several sequence types. In conclusion, this study characterized L. garvieae isolated from mastitis and detected potential adaptations of L. garvieae to various hosts.

**IMPORTANCE** This study provides important genomic insights into a bovine mastitis pathogen, Lactococcus garvieae. Comprehensive genomic analyses of L. garvieae from dairy farms have not been reported. This study is a detailed and comprehensive report of novel features of isolates of L. garvieae, an important but poorly characterized bacterium, recovered in the past 5 years in 6 Chinese provinces. We documented diverse genetic features, including predominant sequence type ST46 and 13 novel MLSTs. Lactococcus garvieae had 6,310 genes, including 1,015 core, 3,641 accessory, and 1,654 unique genes. All isolates had virulence genes coding for collagenase, fibronectin-binding protein, glyceraldehyde-3-phosphate dehydrogenase, superoxide dismutase, and NADH oxidase and resistance to chloramphenicol and clindamycin. Most isolates had *lsaD* and *mdtA* antimicrobial resistance genes. However, no gene was significantly associated with host specificity. This is the first report that characterized L. garvieae isolates from bovine mastitis and revealed potential host adaptations of L. garvieae to various hosts.

## INTRODUCTION

Bovine mastitis is a prevalent and costly disease on dairy farms worldwide (1, 2). It is a multifactorial disease, often caused by bacteria (2). Bacterial pathogens associated with bovine mastitis are broadly classified as major pathogens (Staphylococcus aureus, Escherichia coli, Klebsiella spp., Streptococcus agalactiae, Streptococcus dysgalactiae, Streptococcus uberis, *Enterococcus* spp., etc.) and minor pathogens (non-*aureus* staphylococci, *Lactococcus* spp., *Corynebacterium* spp., etc.) (3). Most studies have focused on major pathogens, with only limited studies of minor pathogens.

Lactococcus garvieae is a zoonotic pathogen reported to cause infections in fish (4) and humans (5–8). It is also considered a minor pathogen for bovine mastitis, with transmission attributed to environmental reservoirs (2). There are limited reports regarding bovine mastitis caused by L. garvieae (9–15)—primarily descriptive studies of phenotypes or genotypes. However, detailed whole-genome characterization of L. garvieae associated with bovine mastitis is lacking.

Predominant strain types have been described for various mastitis pathogens (16–20). Elucidating population structure and diversity of mastitis pathogens informs evidence-based mastitis control programs that target those prevalent strain types.

Bacterial pathogenicity is primarily determined by virulence genes: some facilitate adhesion and invasion, whereas antimicrobial resistance (AMR), particularly multidrug resistance, is an important threat to public health (21). For L. garvieae, several virulence genes and antimicrobial resistance genes were identified using traditional methods (e.g., PCR) targeted at specific virulence genes (22–24). However, a comprehensive profiling of its virulence and antimicrobial resistance genes is lacking.

Host adaptation of bovine mastitis-associated pathogens has been reported for *Staph. aureus* (25) and *Strep. agalactiae* (26). Infections caused by L. garvieae in humans, fish, and cattle have been reported, but potential host adaptation of L. garvieae has not been studied. Therefore, with regard to L. garvieae, our objectives were to (i) resolve the population structure, (ii) identify virulence genes and antimicrobial resistance genes, and (iii) determine genes associated with host specificity.

## RESULTS

A total of 39 L. garvieae isolates were derived from 2,899 clinical mastitis composite milk samples collected from 13 large dairy farms in 6 provinces in North China from April 2017 to September 2021. Detailed information of these isolates is provided in Table 1. In addition, 47 high-quality L. garvieae assemblies from NCBI were included in our survey (see Table S1 in the supplemental material).

**TABLE 1:** Lactococcus garvieae isolates (*n* = 39) recovered from bovine mastitis from 2,899 bovine mastitis (CM) milk samples collected from farms in China

Isolate	Province	Farm	Date (day/mo/yr)
Shanxi-A-01	Shanxi	A	29/1/18
Hebei-B-02	Hebei	B	9/4/18
Hebei-C-03	Hebei	C	11/5/18
InnerMongolia-D-04	Inner Mongolia	D	26/6/18
Hebei-E-05	Hebei	E	16/7/18
InnerMongolia-F-06	Inner Mongolia	F	25/6/19
InnerMongolia-F-07	Inner Mongolia	F	25/6/19
Hebei-G-08	Hebei	G	29/6/19
Hebei-G-09	Hebei	G	29/6/19
Hebei-G-10	Hebei	G	29/6/19
Gansu-H-11	Gansu	H	12/8/20
Gansu-H-12	Gansu	H	12/8/20
Ningxia-I-13	Ningxia	I	12/8/20
Ningxia-I-14	Ningxia	I	12/8/20
Ningxia-I-15	Ningxia	I	12/8/20
Ningxia-I-16	Ningxia	I	12/8/20
Ningxia-I-17	Ningxia	I	24/8/20
Ningxia-I-18	Ningxia	I	24/8/20
Ningxia-I-19	Ningxia	I	24/8/20
Ningxia-I-20	Ningxia	I	24/8/20
Hebei-B-21	Hebei	B	27/8/20
Hebei-B-22	Hebei	B	27/8/20
Ningxia-I-23	Ningxia	I	2/10/20
Shanxi-J-24	Shanxi	J	1/11/20
Shanxi-J-25	Shanxi	J	1/11/20
Ningxia-I-26	Ningxia	I	6/11/20
Ningxia-I-27	Ningxia	I	6/11/20
Ningxia-I-28	Ningxia	I	29/1/21
Shandong-K-29	Shandong	K	9/6/21
Shandong-K-30	Shandong	K	9/6/21
Hebei-L-31	Hebei	L	10/6/21
Hebei-L-32	Hebei	L	10/6/21
Hebei-L-33	Hebei	L	10/6/21
Hebei-L-34	Hebei	L	10/6/21
Hebei-L-35	Hebei	L	10/6/21
Shandong-M-36	Shandong	M	28/6/21
Shandong-M-37	Shandong	M	28/6/21
Hebei-B-38	Hebei	B	12/7/21

### MLST and minimum spanning tree.

**FIG 1:**
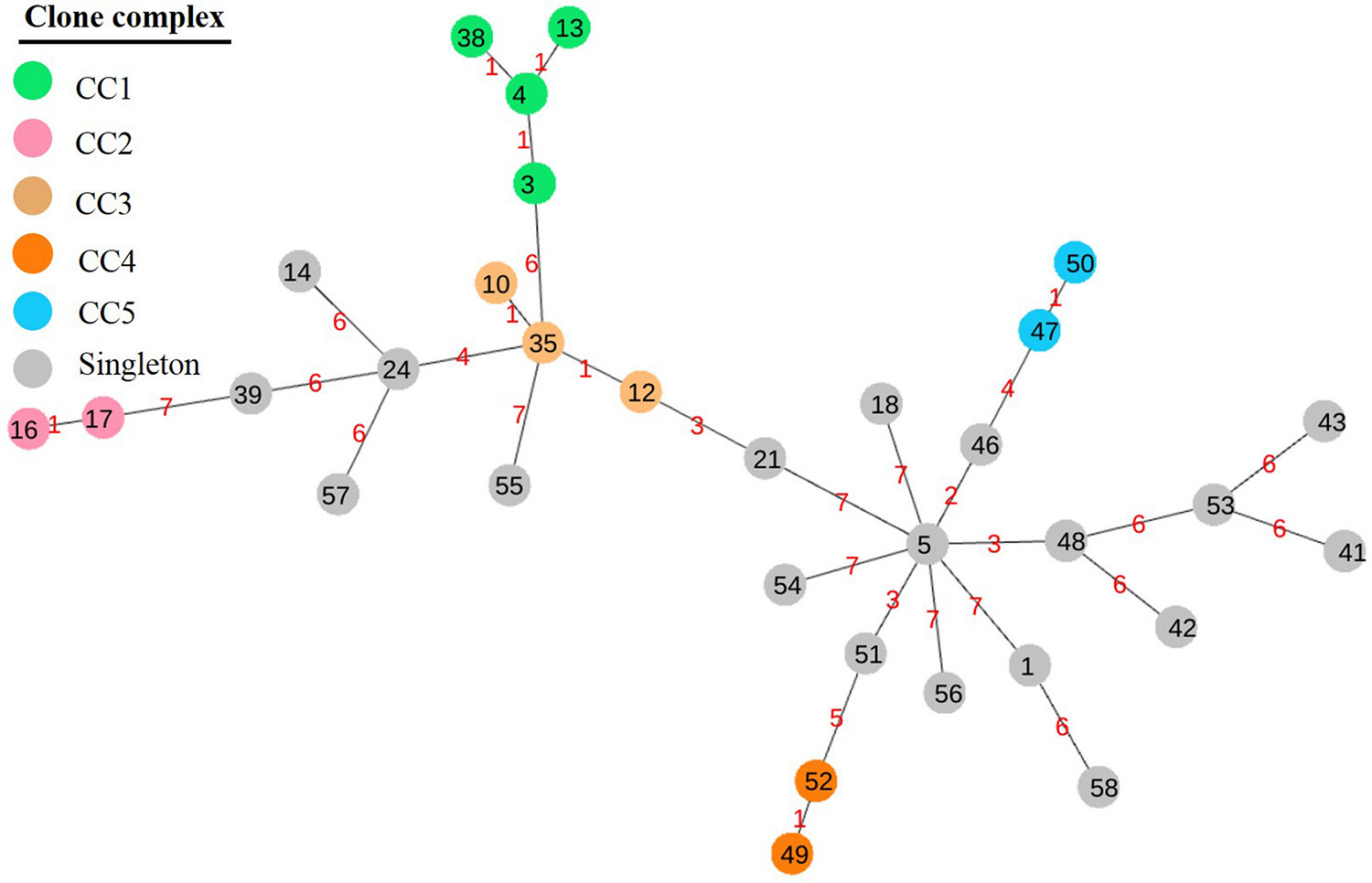
Minimum spanning tree based on multilocus sequence typing for 86 Lactococcus garvieae isolates involving 32 sequence types performed by geoBURST algorithm and visualized by PhyloViz. Five clonal complexes (CC1 to -5) were clustered with similar STs (5 to 7 shared alleles); the number between nodes indicates the number of distinct alleles within them.

Multilocus sequence type (MLST) analysis assigned the 86 L. garvieae isolates into 32 sequence types (STs) (Table 2), of which 13 isolates were novel STs (i.e., ST46 to -58). The most common sequence type was ST46 (*n* = 13), followed by ST48 (*n* = 9). The 32 STs were grouped into 5 clonal complexes (CCs) and 18 singletons: ST3, -4, -13, and -38 into CC1; ST16 and -17 into CC2; ST10, -12, -21, and -35 into CC3; ST49 and -52 into CC4; and ST47 and -50 into CC 5 (Fig. 1). Among the 39 isolates, 6 and 9 isolates formed CC4 and CC5, respectively, whereas the remaining 24 isolates were singletons.

**TABLE 2:** Allelic profiles, sequence types, and clonal complexes of 86 Lactococcus garvieae isolates^*a*^

Isolate ID	ST	CC	Allelic profile						
			*als*	*galP*	*gapC*	*gyrB*	*atpA*	*rpoC*	*tuf*
ATCC 43921	ST1	Sing	1	1	1	1	1	1	1
CCUG32208T	ST1	Sing	1	1	1	1	1	1	1
DSM20684	ST1	Sing	1	1	1	1	1	1	1
FDAARGOS_929	ST1	Sing	1	1	1	1	1	1	1
NBRC100934	ST1	Sing	1	1	1	1	1	1	1
IBB3403	ST3	CC1	3	3	2	3	3	3	3
INF126	ST3	CC1	3	3	2	3	3	3	3
Tac2	ST3	CC1	3	3	2	3	3	3	3
UBA11300	ST3	CC1	3	3	2	3	3	3	3
1001287H_170206_H11	ST4	CC1	3	3	2	3	3	3	4
IPLA31405	ST4	CC1	3	3	2	3	3	3	4
LG728	ST4	CC1	3	3	2	3	3	3	4
MGBC116427	ST4	CC1	3	3	2	3	3	3	4
UBA5784	ST4	CC1	3	3	2	3	3	3	4
I113	ST5	Sing	4	4	3	4	4	4	5
21881	ST10	CC3	9	9	4	7	7	9	3
FDAARGOS_1002	ST10	CC3	9	9	4	7	7	9	3
M14	ST10	CC3	9	9	4	7	7	9	3
TB25	ST12	CC3	9	9	2	7	7	10	3
LG9	ST13	CC1	10	3	2	3	3	3	4
UNIUD074	ST13	CC1	10	3	2	3	3	3	4
8831	ST14	Sing	11	8	2	9	6	11	10
PAQ102015-99	ST14	Sing	11	8	2	9	6	11	10
ATCC 49156	ST16	CC2	12	12	6	10	8	13	6
JJJN1	ST17	CC2	12	12	7	10	8	13	6
Lg2	ST17	CC2	12	12	7	10	8	13	6
LG791	ST17	CC2	12	12	7	10	8	13	6
A1	ST18	Sing	13	13	8	11	9	14	12
DCC43	ST18	Sing	13	13	8	11	9	14	12
FDAARGOS_893	ST18	Sing	13	13	8	11	9	14	12
KS1546	ST21	Sing	9	9	10	7	11	10	3
Hebei-B-22	ST24	Sing	17	16	2	7	12	9	3
Lg-ilsanpaik-gs201105	ST24	Sing	17	16	2	7	12	9	3
FDAARGOS_1062	ST35	CC3	9	9	2	7	7	9	3
Lg-Granada	ST38	CC1	3	23	2	3	3	3	4
TILSE2	ST39	Sing	5	24	2	23	17	19	6
TILSE6	ST39	Sing	5	24	2	23	17	19	6
CT2	ST41	Sing	25	25	9	25	10	21	21
DM12426	ST41	Sing	25	25	9	25	10	21	21
DB24910	ST42	Sing	26	25	13	20	18	22	22
FISHB	ST43	Sing	27	26	9	26	19	23	23
Gansu-H-11	ST46	Sing	29	4	3	27	4	4	5
Gansu-H-12	ST46	Sing	29	4	3	27	4	4	5
Hebei-B-02	ST46	Sing	29	4	3	27	4	4	5
Hebei-B-21	ST46	Sing	29	4	3	27	4	4	5
Hebei-B-38	ST46	Sing	29	4	3	27	4	4	5
Hebei-C-03	ST46	Sing	29	4	3	27	4	4	5
InnerMongolia-F-06	ST46	Sing	29	4	3	27	4	4	5
InnerMongolia-F-07	ST46	Sing	29	4	3	27	4	4	5
Ningxia-I-14	ST46	Sing	29	4	3	27	4	4	5
Shandong-K-29	ST46	Sing	29	4	3	27	4	4	5
Shandong-K-30	ST46	Sing	29	4	3	27	4	4	5
Shanxi-J-24	ST46	Sing	29	4	3	27	4	4	5
Shanxi-J-25	ST46	Sing	29	4	3	27	4	4	5
Hebei-E-05	ST47	CC5	29	27	3	27	4	24	14
Ningxia-I-16	ST47	CC5	29	27	3	27	4	24	14
Ningxia-I-17	ST47	CC5	29	27	3	27	4	24	14
Ningxia-I-23	ST47	CC5	29	27	3	27	4	24	14
Ningxia-I-28	ST47	CC5	29	27	3	27	4	24	14
Ningxia-I-13	ST48	Sing	4	4	3	20	4	24	14
Ningxia-I-15	ST48	Sing	4	4	3	20	4	24	14
Ningxia-I-18	ST48	Sing	4	4	3	20	4	24	14
Ningxia-I-19	ST48	Sing	4	4	3	20	4	24	14
Ningxia-I-20	ST48	Sing	4	4	3	20	4	24	14
Ningxia-I-26	ST48	Sing	4	4	3	20	4	24	14
Ningxia-I-27	ST48	Sing	4	4	3	20	4	24	14
Shandong-M-36	ST48	Sing	4	4	3	20	4	24	14
Shanxi-A-01	ST48	Sing	4	4	3	20	4	24	14
Hebei-B-39	ST49	CC4	30	28	3	28	21	4	18
Hebei-L-31	ST49	CC4	30	28	3	28	21	4	18
Hebei-L-32	ST49	CC4	30	28	3	28	21	4	18
Hebei-L-33	ST49	CC4	30	28	3	28	21	4	18
Hebei-L-35	ST49	CC4	30	28	3	28	21	4	18
Hebei-G-08	ST50	CC5	29	29	3	27	4	24	14
Hebei-G-09	ST50	CC5	29	29	3	27	4	24	14
Hebei-G-10	ST50	CC5	29	29	3	27	4	24	14
Hebei-L-34	ST50	CC5	29	27	3	27	4	24	14
InnerMongolia-D-04	ST51	Sing	31	4	3	4	14	4	14
Shandong-M-37	ST52	CC4	31	28	3	28	21	4	18
122061	ST53	Sing	32	26	14	28	10	25	14
EP01	ST54	Sing	33	Ab	15	29	22	26	24
FDAARGOS_1063	ST55	Sing	34	21	16	30	7	27	25
M79	ST56	Sing	35	30	17	31	23	28	26
RTCLI04	ST56	Sing	35	30	17	31	23	28	26
MGYG-HGUT-00230	ST57	Sing	36	6	2	32	24	8	27
lg38	ST58	Sing	37	31	1	33	25	29	28

a CC, clonal complexes; Sing, singleton; ST, sequence type; Ab, absent.

### Antimicrobial resistance profile and genes.

Antimicrobial resistance (AMR) patterns of L. garvieae are listed in Table 3. All isolates were resistant to chloramphenicol and clindamycin. There were also high resistance rates for amikacin (90%), cefpodoxime (82.5%), cefazolin (45%), and gentamicin (37.5%), whereas few isolates (5%) were resistant to erythromycin. Meanwhile, all isolates were susceptible to penicillin, ampicillin, amoxicillin-clavulanic acid, imipenem, ceftiofur, enrofloxacin, and marbofloxacin. Regarding multidrug resistance, 5 isolates were resistant to 3 antimicrobials, 14 to 4 antimicrobials, 12 to 5 antimicrobials, 6 to 6 antimicrobials, 2 to 7 antimicrobials, and 1 to 8 antimicrobials.

**TABLE 3:** MIC of the 15 antimicrobials tested for 39 Lactococcus garvieae isolates isolated from clinical mastitis composite milk samples in China and control strain ATCC 43921

Antimcrobial	MIC (μg/mL)^*a*^										Resistance rate (%)	MIC (μg/mL)	
	0.12	0.25	0.5	1	2	4	8	16	32	64		MIC_50_	MIC_90_
Penicillin		9	7	19	5						0	1	2
Ampicillin		3	9	24	4						0	1	1
Amoxicillin-clavulanic acid				1	4	26	9				0	4	8
Imipenem	11	17	11	1							0	0.25	0.5
Cephalothin						10	26	4			10	8	8
Cefazolin				2	20	11	6	1			45	2	8
Cefpodoxime				2	5	21	12				82.5	4	8
Ceftiofur	16	17	7								0	0.25	64
Amikacin								4	5	31	90	64	64
Gentamicin				1	12	12	15				37.5	4	8
Erythromycin	19	6	13	2							5	0.25	0.5
Clindamycin							1	23	11	5	100	16	32
Enrofloxacin	1	3	17	15	3	1					0	0.5	1
Marbofloxacin			2	20	15	3					0	1	2
Chloramphenicol							18	14	2	6	100	16	64
Vancomycin			15	22	3						0	1	1

a Resistance breakpoints are highlighted by dark gray shading, and intermediate breakpoints are highlighted by light gray shading. Cells without shading indicate that no breakpoints were available in the literature.

**FIG 2:**
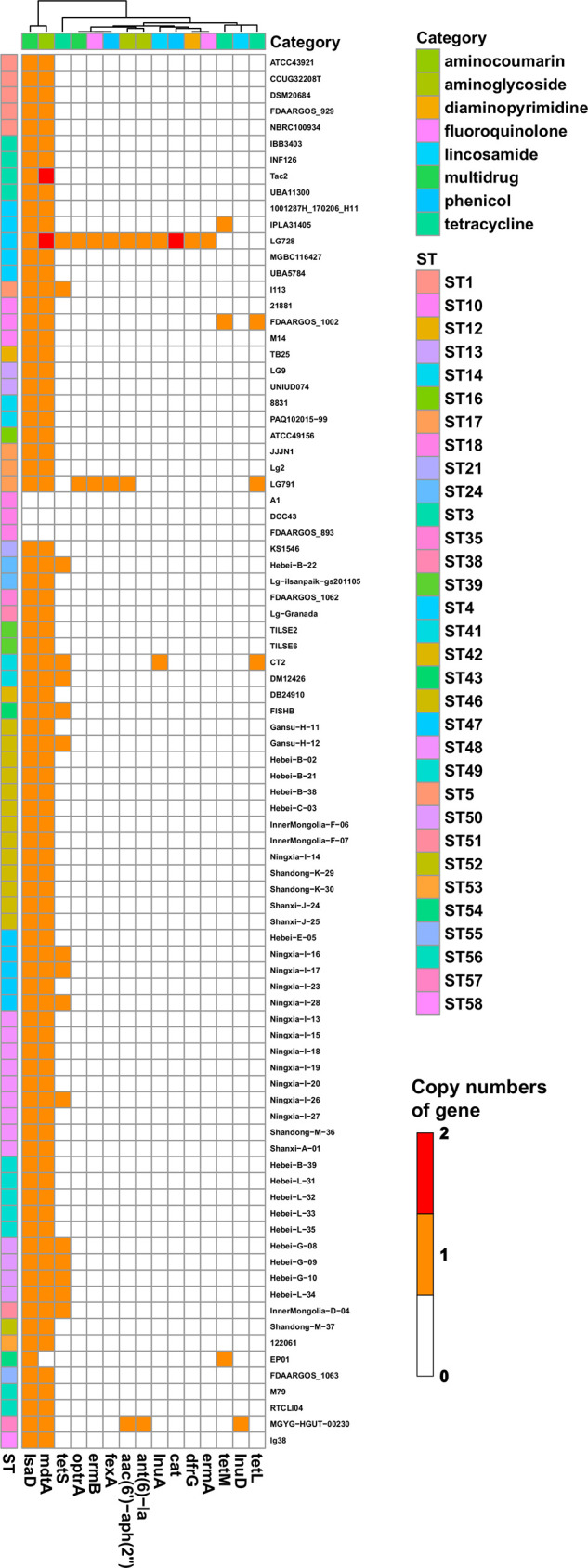
Distribution of antimicrobial resistance genes against each category of antimicrobial resistance in 86 Lactococcus garvieae isolates. A total of 15 AMR genes were identified by Resfinder and CRAD in all 86 Lactococcus garvieae isolates. Copy number (range, 0 to 2) is indicated by white to red. STs are marked with colored bars along the *y* axis. The phylogenetic tree, based on the presence of AMR genes in isolates, is presented as a cladogram in the panel along the *x* axis, as well as 7 AMR categories.

Distributions of AMR genes with country, host, and clonal complex information are presented in Fig. 2. The most common genotypic AMR markers were as follows: (i) multidrug protein first reported in L. garvieae, represented by the *lsaD* gene (97% of isolates); (ii) the resistance-nodulation-cell division antibiotic efflux pump, represented by the *mdtA* gene (97% isolates); and (iii) the tetracycline-resistant ribosomal protection protein, encoded by the *tetS* gene (14 isolates, including 12 from current bovine samples). Two other tetracycline resistance genes, *tetL* and *tetM*, were identified in only 3 isolates. Three isolates, namely, LG728, LG791, and MGYG-HGUT-00230, harbored the most abundant AMR genes, the numbers of which were 14, 7, and 5, respectively. Genes *cat*, *dfrG*, *ermA*, and *lunD* were present only in LG728, whereas gene *lnuD* was found only in MGYG-HGUT-00230. Genes *ermB*, *fexA*, and *optrA* were present in LG728 and LG791. Genes *acc(6′)-aaph(2′′)* and *ant(6)-la* were identified in LG728 and MGYG-HGUT-00230. However, no AMR genes were detected in 3 isolates that belonged to ST18: A1, DCC43, and FDAARGOS_893.

### Virulence genes.

**FIG 3:**
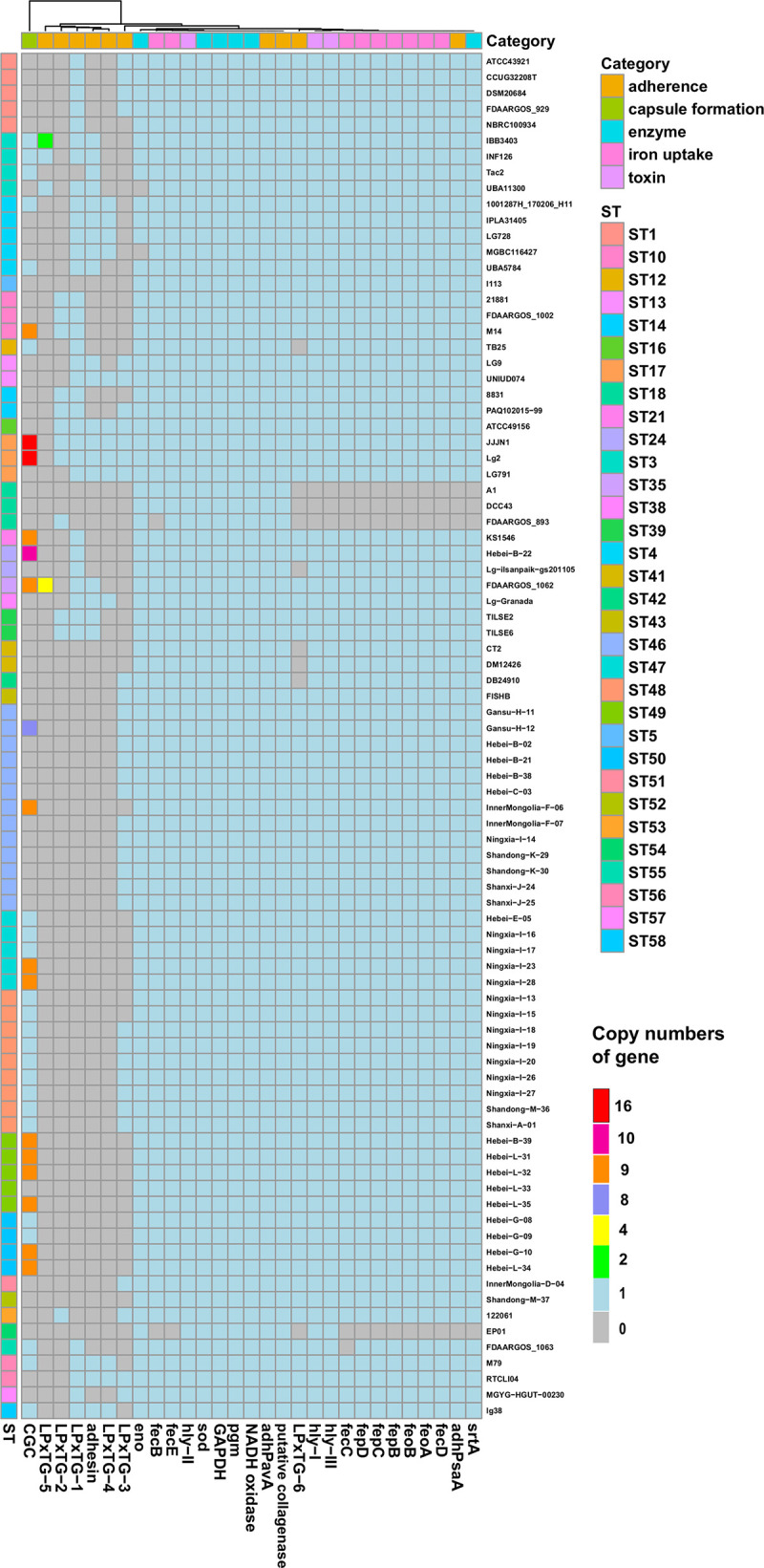
Distribution of virulence genes in 86 Lactococcus garvieae isolates. In total, 29 AMR genes were identified by an integrated Lactococcus garvieae virulence gene database in all 86 Lactococcus garvieae isolates. The copy number ranged from 0 to 16. STs are marked with colored bars along the *y* axis. The phylogenetic tree, based on the presence of virulence genes in isolates, is presented as a cladogram in the panel along the *x* axis, as well as 5 virulence gene categories.

The occurrence and distribution of putative virulence genes are shown in Fig. 3, with detailed information provided in Table S2 and Text S1 in the supplemental material. The putative virulence genes were classified into 5 functional categories: toxin, iron uptake, capsule formation, adherence, and enzyme.

Among the 3 toxin-related genes, *hly-II* was present in all isolates, whereas *hly-I* and *hly-III* were absent in ST18 only (represented by 3 isolates: A1, DCC43, and FDAARGOS_893). Nine iron uptake genes (*fepB*, *fepC*, *fepD*, *fecB*, *fecC*, *fecD*, *fecE*, *feoA*, and *feoB*) were detected in >81 isolates. In contrast, *fecE* was absent only in EP01. However, *fecB* was absent in EP01 and FDAARGOS_893*. fecD*, *feoA*, *feoB*, *fepB*, *fepC*, and *fepD* were absent in 4 isolates: A1, EP01, DCC43, and FDAARGOS_893. Furthermore, *fecC* was absent in 5 isolates: A1, EP01, DCC43, FDAARGOS_893, and FDAARGOS_1063.

With respect to the 10 adherence-associated virulence genes, *adhPavA* and putative collagenase were identified in all isolates. The gene *adhPsaA* was absent in the same 4 isolates mentioned above (A1, EP01, DCC43, and FDAARGOS_893), whereas the adhesin gene was detected in 22 isolates. Genes expressing LPxTG proteins (LPxTG-1, LPxTG-2, LPxTG-3, LPxTG-4, LPxTG-5, and LPxTG-6) were found in 4% to 43% of isolates.

Six enzyme-related virulence genes were detected in almost all isolates, although *eno* and *srtA* were exclusively absent from 2 isolates (MGBC116427 and UBA11300) and 4 isolates (A1, EP01, DCC43, and FDAARGOS_893), respectively. Regarding 16 genes encoding the capsule gene cluster, the number of genes present in isolates varied from 0 to 16. Both Lg2 and JJJN1 had all 16 genes, whereas 47 isolates had none.

**FIG 4:**
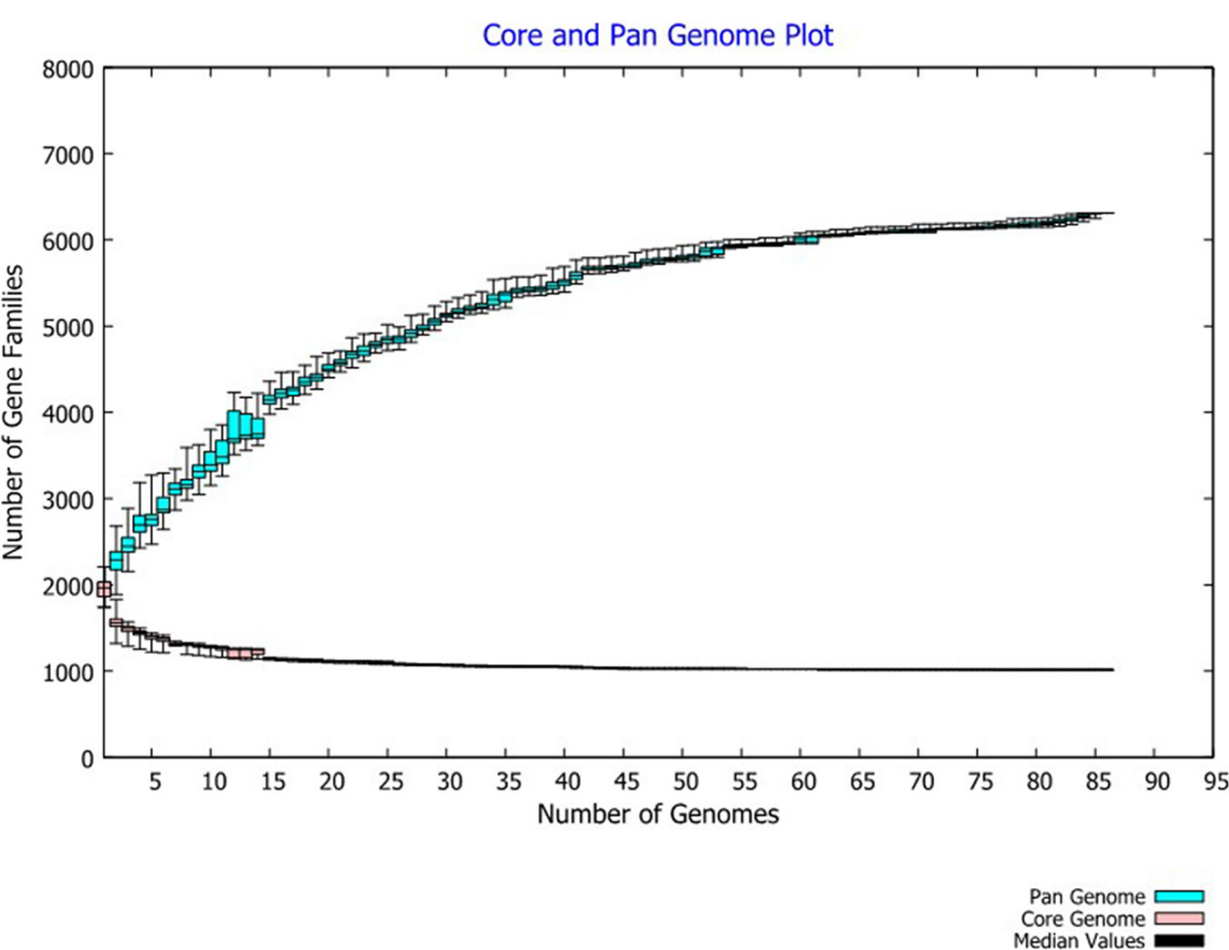
Pangenome of 86 Lactococcus garvieae isolates in this study. The pangenome consisted of 6,310 genes: the size of the genome in the pangenome increased as the number of isolates increased, but pangenome size approached convergence. The number of core genes (shared by all isolates) was relatively constant at 1,015 genes.

Co-occurrence of virulence genes is visualized in Fig. 4, where each box includes a phi coefficient value. A phi value of 1.0 indicates a perfect positive association between the 2 variables, whereas values of >0.7 indicate a fair positive association. Most associations among virulence genes were very weak. However, there were strongly positive associations between 8 genes, including *adhPsaA*, *srtA* and 6 iron uptake genes (*fecD*, *feoA*, *feoB*, *fepB*, *fepC*, and *fepD*). In addition, toxin-related genes *hly-I* and *-III* were also strongly positively associated with each other and those 8 genes. Furthermore, *fecC* was strongly positively associated with *hly-I*, *hly-III*, and the 8 genes. LPxTG-4 was strongly positively associated with the adhesin gene, whereas *fecB* had strong associations with the 8 genes. However, there were no strong negative associations among virulence genes.

### Pangenome analyses.

**FIG 5:**
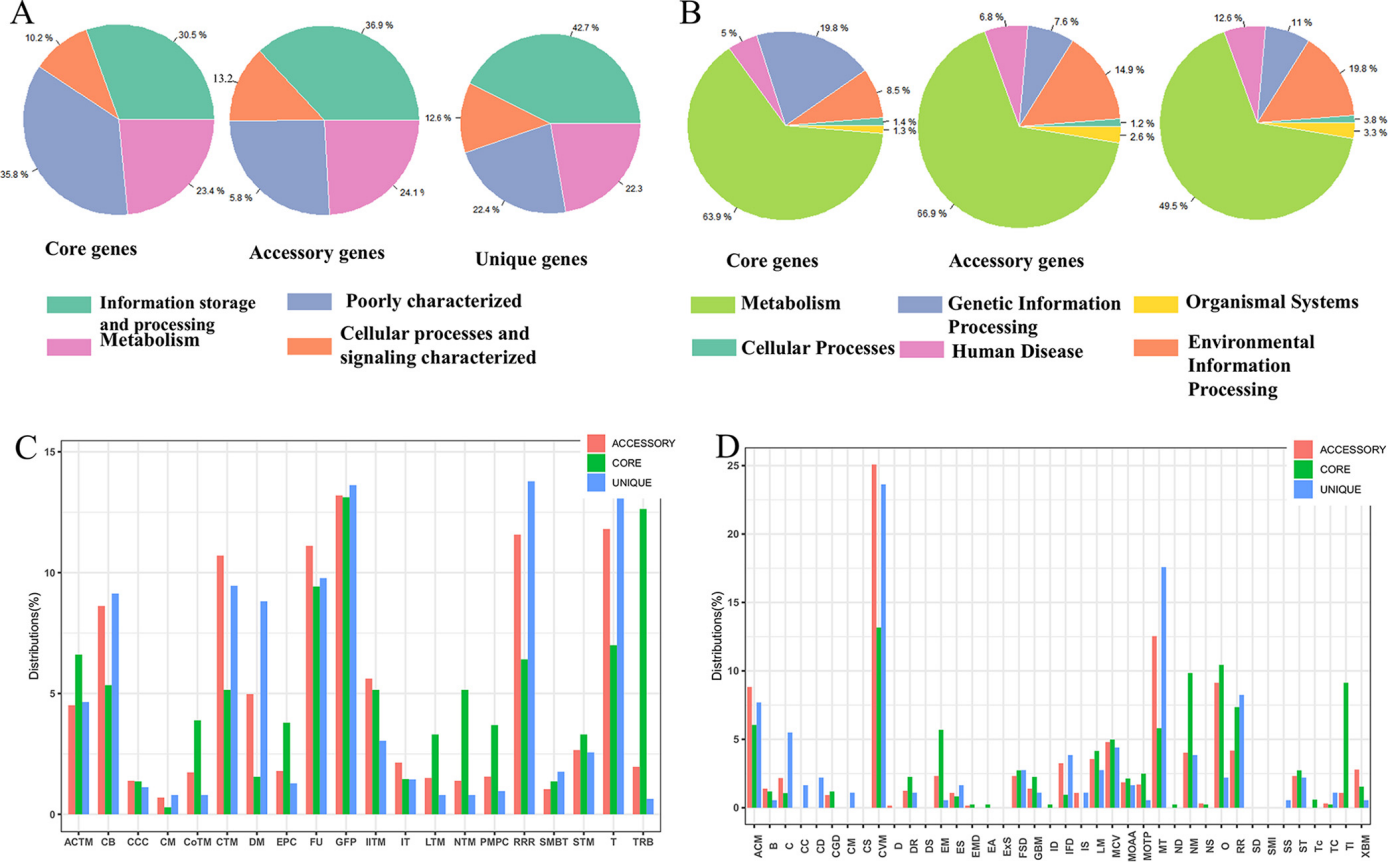
Differential distribution of COG and KEGG functional categories in core, accessory, and unique genes: (A) Proportions of 4 classes of COG functional categories in core, accessory, and unique genes; (B) proportion of 6 classes of KEGG functional categories in core, accessory, and unique genes; (C) COG functional subcategories in core, accessory, and unique genes. CCC, cell cycle control, cell division, chromosome partitioning; CB, cell wall/membrane/envelope biogenesis; CM, cell motility; PMPC, posttranslational modification, protein turnover, and chaperones; STM, signal transduction mechanisms; IT, intracellular trafficking, secretion, and vesicular transport; DM, defense mechanisms; TRB, translation, ribosomal structure, and biogenesis; T, transcription; RRR, replication, recombination, and repair; EPC, energy production and conversion; CTN, carbohydrate transport and metabolism; ACTM, amino acid transport and metabolism; NTM, nucleotide transport and metabolism; CoTM, coenzyme transport and metabolism; LTN, lipid transport and metabolism; SMBT, secondary metabolite biosynthesis, transport, and catabolism; IITM, inorganic ion transport and metabolism; GFP, general function prediction only; FU, function unknown. (D) KEGG functional subcategories in core, accessory and unique genes. ACM, amino acid metabolism; B, biosynthesis of other secondary metabolites; C, cancers; CVM, carbohydrate metabolism; CD, cardiovascular diseases; CGD, cell growth and death; CM, cell motility; CC, cellular community; CS, circulatory system; D, development; DS, digestive system; DR, drug resistance; EMD, endocrine and metabolic diseases; ES, endocrine system; EM, energy metabolism; EA, environmental adaptation; ExS, excretory system; FSD, folding, sorting, and degradation; GBM, glycan biosynthesis and metabolism; ID, immune diseases; IS, immune system; IFD, infectious diseases; LM, lipid metabolism; MT, membrane transport; MCV, metabolism of cofactors and vitamins; MOAA, metabolism of other amino acids; MOTP, metabolism of terpenoids and polyketides; NS, nervous system; ND, neurodegenerative diseases; NM, nucleotide metabolism; O, overview; RR, replication and repair; SS, sensory system; ST, signal transduction; SMI, signaling molecules and interaction; SD, substance dependence; Tc, transcription; Tl, translation; TC, transport and catabolism; XBM, xenobiotic biodegradation and metabolism.

The pangenome of 86 L. garvieae isolates tested in this study had 6,310 genes. The core genome (shared by 100% of isolates) consisted of 1,015 genes. The accessory genome (genes in >2 isolates but not in all isolates) consisted of 3,641 genes, and the unique genome was composed of 1,654 genes. According to BPGA calculation, the pangenome was open but approached convergence (Fig. 4). Functional annotation of genes revealed a distribution of functional categories among 3 pangenome sets according to the COG (Clusters of Orthologous Genes) and KEGG databases (Fig. 5). The proportions of 4 COG categories were consistent in core, accessory, and unique genes (Fig. 5A). The functions of defense mechanisms, transcription and replication, and recombination and repair were enhanced in unique genes, whereas the functions of translation, ribosomal structure, and biogenesis were enhanced in core genes in KEGG functional pathways (Fig. 5B). The COG functional categories enriched in the unique genome included human disease and membrane transport (Fig. 5C). In contrast, COG categories enriched in the core genome included energy metabolism, nucleotide metabolism, and translation (Fig. 5D).

### Phylogenetic analyses.

A phylogenetic tree was constructed based on core genes of 86 L. garvieae genomes (see Fig. S1 in the supplemental material). The longer the branch, the more distant the evolutionary relationship. The tree had 3 clades that contained 4, 38, and 44 isolates, respectively. All current isolates, except Hebei-B-22, were in the same clades. EP01 was phylogenetically distant from all others. Isolates from various hosts had signs of host adaptation.

The phylogenetic tree based on 16S rRNA (Fig. S1) was similar to that using core genes, with only minor differences. For example, LG9 was assigned to clade A in the core gene-based phylogenetic tree but was in clade B in the 16S rRNA-based phylogenetic tree.

Both trees corresponded well with STs and CCs predicted by GrapeTree analysis. For the core gene-based phylogenetic tree (Fig. S2), all isolates that belonged to the same CC were grouped in the same cluster, except 2 isolates (LG9 and IBB3403) in the 16S rRNA-based phylogenetic tree.

### Pangenome-wide association analyses.

No significant association was detected between genes and either country or host by pangenome-wide association analysis.

### Core genome SNP analyses.

**FIG 6:**
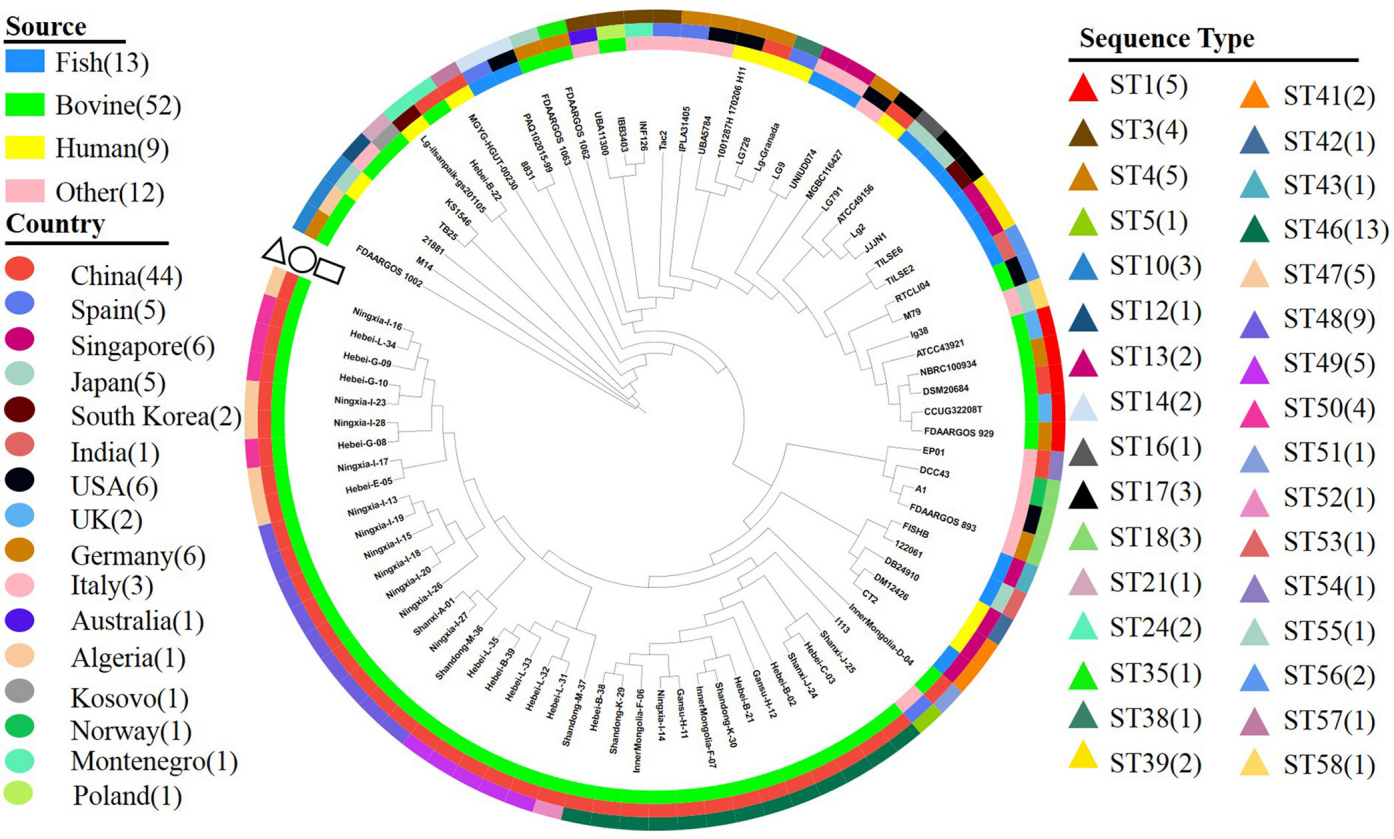
Phylogenetic tree based on core genome single nucleotide polymorphism of 86 Lactococcus garvieae isolates. Shown are the source of the host (4 hosts [the first ring, indicated by rectangles]) and country (16 countries [the second ring, indicated by circles]), as well as sequence types (STs) (32 STs [the outermost ring, indicated by triangles]), of 86 Lactococcus garvieae isolates.

The core genome single nucleotide polymorphism (SNP)-based phylogenetic tree with metadata annotation is displayed in Fig. 6. Numbers of core genome SNPs among 86 isolates are provided in Table S3. Several isolates from various hosts were phylogenetically closely related in core SNPs. For example, the numbers of SNPs between isolates within the same MLST but a different host were 0 for DM12426 (human) and CT2 (fish), 5 for 1001287H_170206_H11 (human) and UBA5784 (metal), 11 for Lg-ilsanpaik-gs201105 (human) and Hebei-B-22 (cow), and 105 for 21881 (human) and M14 (cow), implying potential host adaptation in L. garvieae.

### Associations between co-occurrences of virulence genes.

**FIG 7:**
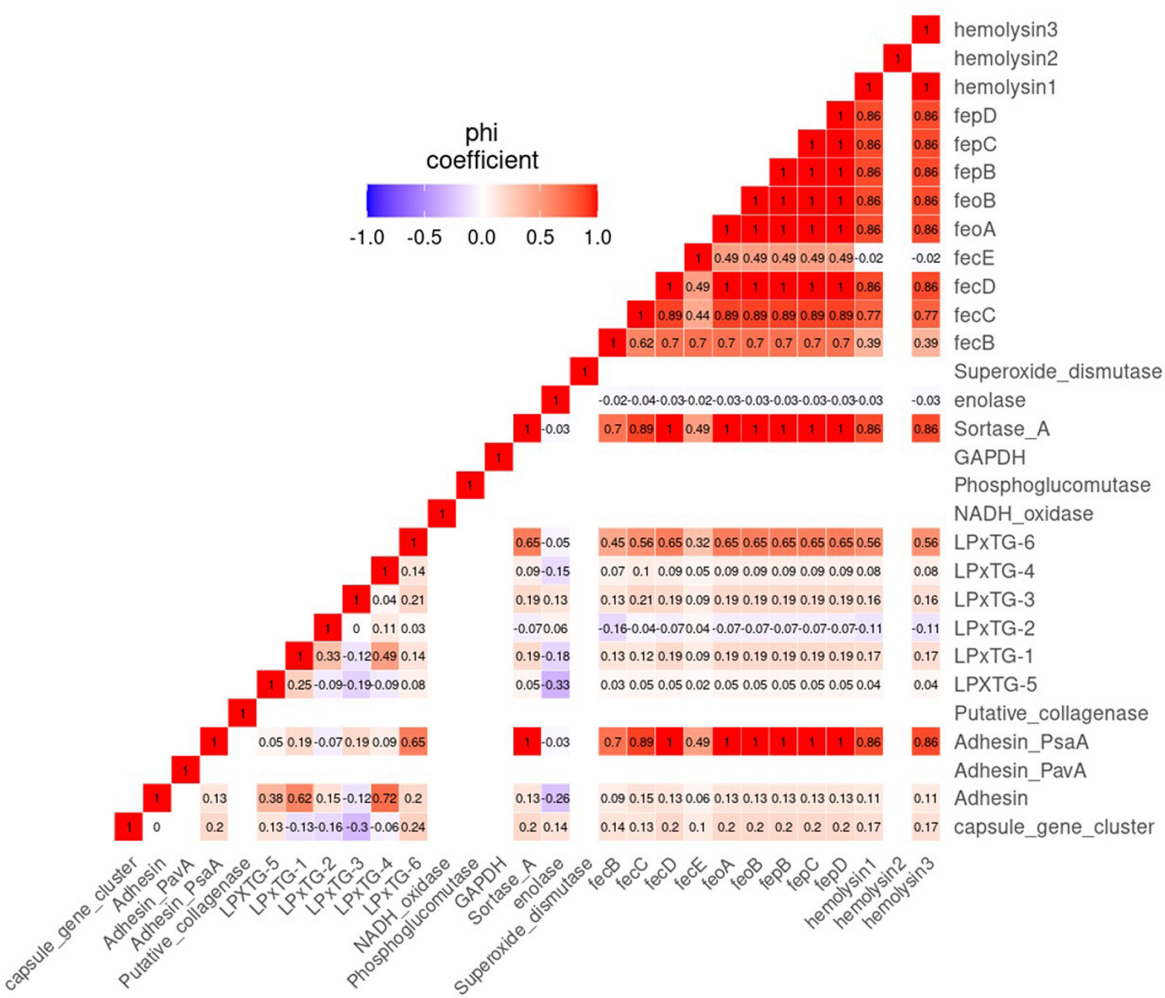
Co-occurrence of virulence genes in 86 Lactococcus garvieae isolates. Associations were computed using the phi coefficient. A phi coefficient has values from −1 to 1, as follows: −1 indicates a perfectly negative relationship between the 2 variables, 0 indicates no association between the 2 variables, and 1 indicates a perfectly positive relationship between the 2 variables. Colors represent type of association: blue, negative; red, positive.

Co-occurrence of virulence genes is visualized in Fig. 7. The LPxTG-6, *srtA*, and *adhPsaA* genes were in co-occurrence with *fecB*, *fecC*, *fecD*, *feoA*, *feoB*, *fepB*, *fepC*, *fepD*, *h1y-I*, and *hly-III*.

## DISCUSSION

Although L. garvieae was first isolated as a causative agent of bovine mastitis (27), most reports have focused on epidemiology of isolates from fish or humans. In addition, many studies used sequencing to investigate genotypic characteristics of L. garvieae isolates (8, 28–40). Therefore, we collected 39 L. garvieae isolates from bovine mastitis in China and conducted comparative genome sequence analyses.

This is apparently the first report of the prevalence of L. garvieae in Chinese dairy herds. The prevalence of L. garvieae from clinical mastitis samples was 1.35% during 2017 to 2021, which increased from 0% in 2017 to 4.10% in 2020 in China. That L. garvieae has been misclassified as Streptococcus spp. (41) has resulted in underreporting. Similarly, the true incidence of human infective endocarditis is difficult to assess due to misidentification with other Gram-positive cocci (42).

The MLST analysis clustered 86 L. garvieae isolates into 32 distinct STs, with 5 CCs and 18 singletons, consistent with the population structure in isolates from other hosts (37, 38, 43), the environment, or food (44). All strains, except Hebei-B-22, were new STs and phylogenetically close to one other. However, they were distant from isolates of bovine mastitis in other countries, which may indicate geographic effects on phylogeny. Meanwhile, new ST profiles were comprised of new alleles in gene loci (e.g., *als*, *gyrB*, and *galP*), perhaps due to a different evolution rate for those loci (38).

Understanding phylogenetic relationships between strains is important for characterizing pathogen transmission. In this study, 3 phylogenetic trees were constructed using core genes, core genome SNPs, and 16S rRNA, respectively. The core gene-based tree and 16S rRNA-based tree produced similar clades, but the 16S rRNA-based tree failed to resolve relationships toward tree tips. Furthermore, a core gene tree was in line with MLST and CC. This was not surprising, as a 16S rRNA tree is based on only 1 gene, representing only a very small portion of the genome. Therefore, many studies have recommended core genes to infer phylogenies (45, 46).

Although the pathogenicity of L. garvieae is poorly understood, some factors associated with pathogenicity have been determined, including the presence of a capsule, hemolytic activity via secreted proteins (47), and production of siderophores (48). In fish, capsulated L. garvieae Lg2 was more virulent than the noncapsulated isolate ATCC 49156 (49). The capsule gene cluster, located in a genomic island, was identified in Lg2 but absent in ATCC 49156: that could be crucial for virulence of L. garvieae in fish (40). However, the capsule gene cluster was not detected in all clinical fish isolates from Japan, Spain, Italy, France, Turkey (22), the United States (39), or India (50) nor in any human isolates (31, 51). In this study, only 2 of 86 isolates had the complete capsule gene cluster, which confirmed that it was not essential for virulence.

Proteases are among the important virulence genes causing rapid and extensive destruction of host tissue. For example, enolase (50) can cleave an extracellular proteinaceous matrix and therefore break a host’s structural barrier during colonization. In addition, hemolysin genes may act with secreted proteases to promote host tissue destruction. Genes encoding biosynthesis of iron uptake may be involved in iron acquisition during host colonization (48). LPxTG protein (Leu-Pro-any-Thr-Gly), an important virulence factor in L. garvieae, binds to the peptidoglycan of cell wall by transpeptidase enzymes called sortases (40). In a previous study, an L. garvieae strain isolated from rainbow trout colonized nonphagocytic cells with the help of LPxTG proteins (52). LPxTG proteins and sortases have important roles binding pathogenic bacteria to their host. In this study, genes coding for adhesin, proteases, hemolysin, iron uptake, and LPxTG protein were detected in most or all isolates. Strong positive associations within LPxTG-4, *adhPsaA*, *srtA*, and iron uptake genes suggested they may act together to promote host tissue destruction and colonization. Furthermore, the gene *pgm*, identified in all isolates, produces protein with an important role in antibody production (53).

MIC results were consistent with reports that L. garvieae isolates from dairy farms were susceptible to penicillin, ampicillin, amoxicillin-clavulanic acid, imipenem, ceftiofur, enrofloxacin, vancomycin, and marbofloxacin (10, 54). However, compared to a previous report (10), there were variable degrees of increasing resistance rates for 8 antibiotics, including clindamycin (94% to 100%), chloramphenicol (6.4% to 100%), amikacin (2.1% to 90%), cefpodoxime (0% to 83%), cephalothin (0% to 10%), cefazolin (40% to 45%), gentamicin (0% to 38%), and erythromycin (0% to 5%). High resistance of clindamycin has been described as intrinsic for L. garvieae and proposed as a selection criterion to distinguish between L. garvieae and Lactococcus lactis (55). The *lsaD* gene, identified in most isolates (83/86), could be responsible for intrinsic resistance. *lsaD* is a novel *lsa*-type family gene detected in lincomycin-resistant strains isolated from fish (56). The *lsa*-type genes are responsible for cross-resistance to lincosamides, streptogramins, or pleuromutilins [here referred to as the LSA(P)-resistant phenotype], by coding ATP-binding cassette F proteins in Gram-positive pathogens, including staphylococci (57), streptococci (58), enterococci (59), and lactococci (56). Increasing resistance against cephalosporins may be related to increasing use of these antibiotics on Chinese dairies (60).

The multidrug transporter *mdtA* is another AMR gene present in most isolates. This gene originally conferred resistance to macrolides, lincosamides, streptogramins, and tetracycline in L. lactis (61), but mutations in the C-motifs of *mdtA* from L. garvieae confer susceptibility to erythromycin and tetracycline (54). Furthermore, all 39 isolates with *mdtA* had limited resistance to erythromycin (5%). Some isolates (16/86) harbor the *tetS* gene, including 10 current isolates. Three isolates from the human gut in China (LG729, LG729, and MGYG-HGUT-00230) contained the most abundant AMR genes. Notably, 9 AMR gene were only present in the 3 isolates, including *cat*, *dfrG*, *ermA*, *ermB*, *lunD*, *fexA*, *optrA*, *acc(6′)-aaph(2′′)*, and *ant(6)-la*. The *optrA* gene, first identified in enterococci, has been reported in staphylococci, streptococci, Clostridium perfringens, and Campylobacter coli; it confers resistance to oxazolidinones and phenicol and was identified on a plasmid of L. garvieae (62).

The spread of antibiotic resistance genes in bacterial populations is aided by various mechanisms of horizontal gene transfer, with plasmid-mediated transfer being the main mechanism for transmission of resistance genes (63). Horizontal gene transfer between bacteria is largely mediated by specialized mobile genetic elements, including plasmids, bacteriophages, transposon, insert sequences (ISs), integrons, etc., and has been reported in L. garvieae. Both *tetS* and *tetM* were associated with conjugative transposon-associated genes in isolates from healthy fish intestines (64). Most of the ISs in L. garvieae had substantial homology to Lactococcus lactis elements, implying movement of ISs between these 2 species that are phylogenetically closely related (65, 66). That these 9 AMR genes were reported only in humans does not support the assertion that AMR genes are transferred to humans from fish or dairy products. Regardless, L. garvieae could be a reservoir for antibiotic resistance genes for other bacteria.

In this study, no genes were associated with host specificity, consistent with the phylogenetic analysis and the core genome SNP analysis that host adaptation occurs in L. garvieae isolates. Previous research (5) summarized human L. garvieae infections associated with consumption of raw fish, seafood, or unpasteurized milk. The core genome SNP analysis underlies potential host adaptation of L. garvieae. Meanwhile, adhesins, hemolysin, fibronectin-binding proteins, penicillin acylase and WxL domain-containing proteins are considered to actively promote bacterial colonization (67); most had high similarity across hosts in those coding sequences. Regardless, the underlying mechanisms remain unclear. Consequently, further studies are needed to determine host adaptation mechanisms of L. garvieae.

### Conclusions.

This was apparently the first comparative genomic analysis of L. garvieae isolates from bovine mastitis in China. The incidence of L. garvieae mastitis was 1.35% in China. Most isolates (38/39) were novel sequence types, 3 antimicrobial resistance genes (*mdtA*, *lsaD*, and *tetS*) were identified, and there was evidence of host adaptation.

## MATERIALS AND METHODS

### Statement of ethics.

This study was conducted in accordance with ethical guidelines and standard biosecurity and institutional safety procedures of China Agricultural University (CAU) (Beijing, China). Ethical approval was not needed, as no animal study was involved.

### Sample collection and bacterial identification.

Milk samples from cases of clinical mastitis were collected aseptically from dairy cows on Chinese dairy farms and sent to the Mastitis Diagnostic Laboratory at the College of Veterinary Medicine, CAU, Beijing, China. Pathogens were identified by bacteriological culture, colony morphology, and 16S rRNA sequencing, according to National Mastitis Council guidelines (68). In brief, 50 μL milk was spread on tryptone soy agar with 5% defibrinated sheep blood. The plate was incubated aerobically at 37°C for 24 h. Bacterial colony morphology was recorded; samples with ≥3 morphologically distinct colonies were considered contaminated and excluded from subsequent analyses.

### Antimicrobial susceptibility testing.

For all 39 L. garvieae isolates, MIC of 15 antimicrobials (Chinese National Institutes for Food and Drug Control, Beijing, China), commonly used to treat clinical mastitis in China, were determined by the broth microdilution method, according to the Clinical and Laboratory Standards Institute (CLSI) guidelines VET01-A4 (CLSI, 2013), with reported breakpoints (10). Staphylococcus aureus ATCC 29213 was used as the quality control strain.

### Genome assembly and annotation.

Genomic DNA of putative isolates was extracted using a bacterial DNA extraction kit (TransGen Biotech, Beijing, China) according to the manufacturer’s instruction. Extracted DNA was quantified with a NanoDrop One spectrophotometer (Thermo Fisher Scientific, Waltham, MA, USA) prior to 16S rRNA gene sequencing (Beijing Sunbiotech, Inc., Beijing, China). Whole-genome DNA was paired-end sequenced (2 × 150 bp) using Illumina NovaSeq 6000 (Illumina, San Diego, CA, USA) at Shanghai Personal Biotechnology Co., Ltd. (Shanghai, China). For raw reads, quality control was done with FastQC version 0.11.9 (https://github.com/s-andrews/FastQC). Low-quality bases were trimmed using fastp version 0.20.1 (https://github.com/OpenGene/fastp) with default settings. Quality trimmed reads were assembled into scaffolds using SPades version 3.13.1 (https://github.com/ablab/spades) with auto coverage cutoff and shovill version 1.1.0 (https://github.com/tseemann/shovill) with default settings. Thereafter, 2 assembled scaffolds for each isolate were obtained, and a draft genome of each isolate was selected using Quast version 5.0.2 (https://github.com/ablab/quast) with *N*_50_, and *L*_50_ from the above-mentioned 2 assembled scaffolds. Assembly completeness was assessed using Busco version 5.2.2 (https://github.com/WenchaoLin/BUSCO-Mod) with reference to lineage lactobacillales_odb10. Only genomes with completion ≥ 95% were considered a “high-quality draft genome” and were included in further analyses (69). In addition, whole-genome sequence assembly fasta files of 51 L. garvieae (accessed on 24 March 2022) were downloaded from NCBI. To ensure high-quality genomes, all genomes were analyzed by BusCom version 5.2.2 (lineage lactobacillales_odb10) and 3 assemblies were excluded from subsequent analyses. There were 3 ATCC 49156 assemblies; we chose the one with the highest assembly level. Therefore, a total of 39 isolates from composite (or quarter) milk samples and 47 assemblies from NCBI were obtained in the subsequent genome annotation and pangenome analysis. Annotation of the genome was performed using Prokka version 1.14.6 (https://github.com/tseemann/prokka) with default settings.

### MLST analyses.

Multilocus sequence typing using whole-genome sequences was performed to determine sequence types (STs) of the 86 isolates. An L. garvieae MLST database was constructed based on reported data sets (51) as there was no publicly available MLST scheme for L. garvieae. Similarly, the database was integrated into the ABRicate local database, and the 86 L. garvieae genomes were aligned against the data set by ABRicate. Sequence types were assigned to new allele patterns and added to the existing L. garvieae MLST scheme (51). The clonal complex (CC) was defined as a group of STs in which every ST shared at least 5 of 7 identical allele profiles with at least 1 other ST in the group. The minimum spanning tree (MST) was constructed by the goeBURST algorithm and visualized with the PhyloViz web server (https://online.phyloviz.net/index) to infer phylogenetic relationships among STs.

### Identification of antimicrobial resistance genes and virulence genes.

Antimicrobial resistance genes were identified by BLAST analysis L. garvieae genomes against ResFinder database via Resfinder version 4.1.11 (https://bitbucket.org/genomicepidemiology/resfinder.git) and the Comprehensive Antibiotic Resistance Database (https://card.mcmaster.ca/) via RGI version 5.2.1 (https://github.com/arpcard/rgi). A set of virulence genes of L. garvieae was summarized from previous reports (22, 51, 70): they included genes associated with hemolysins I, II, and III (*hlyI*,-*II* and *-III*), iron uptake (*fepB*, *fepC*, *fepD*, *fecB*, *fecC*, *fecD*, *fecE*, *feoA*, and *feoB*), the capsule gene cluster (CGC), adhesion (the adhesin gene, *adhPavA*, and *adhPsaA*), putative collagenase, LPxTG surface proteins 1, 2, 3, 4, 5, and 6 (LPxTG-1, LPxTG-2, LPxTG-3, LPxTG-4, LPxTG-5, and LPxTG-6), and enzyme-related virulence genes NADH oxidase, glyceraldehyde-3-phosphate dehydrogenase (GAPDH), phosphoglucomutase (*pgm*), superoxide dismutase (*sod*), enolase (*eno*), and sortase A (*srtA*). The database was integrated into the ABRicate local database. All 86 (39 from our current study and 47 from NCBI) L. garvieae genomes were BLAST analyzed against the database using ABRicate to determine virulence genes. The presence of antimicrobial resistance or virulence gene was defined using cutoff values of 80% sequence coverage and 80% nucleotide identity (ABRicate default settings).

### Pangenome analyses.

The pangenome of 86 L. garvieae isolates was computed using BPGA version 1.3 (https://iicb.res.in/bpga/) with USEARCH algorithm to cluster orthologous gene families using faa files of local isolates produced by Prokka and retrieved from NCBI. For BPGA, a core gene was defined as a gene present in all the genomes, an accessory gene was present in >1 genome but not all genomes, and a unique gene was only present in a single genome. Functional annotations of core, accessory, and unique genes were obtained after comparing sequences to COG and KEGG databases incorporated in BPGA version 1.3.

### Phylogenetic analyses.

A 16S rRNA phylogenetic tree was constructed based on 16S rRNA genes. In addition, we also constructed another phylogenetic tree using alignment of core genes produced by BPGA. For 16S rRNA phylogenetic analysis, Barrnap version 0.9 (https://github.com/tseemann/barrnap) was used to extract 16S rRNA genes from the whole-genome sequence. The 16S rRNA gene sequences were edited and aligned using the MAFFT multiple-sequence alignment algorithm (stargety “L-IINS-I”; https://github.com/GSLBiotech/mafft). Maximum likelihood (ML) trees based on this alignment were constructed using FastTree version 2.1 (https://github.com/PavelTorgashov/FastTree). Visualization of the phylogenetic tree was performed using iTOL (https://itol.embl.de/) with isolate metadata (clonal complex, source of host, and country).

### Pangenome-wide association analyses.

To identify genes potentially associated with traits (e.g., host, clonal complex, and country), pangenome-wide association analysis was performed using Scoary. Annotation of whole-genome sequences of the 86 L. garvieae isolates were performed using Prokka version 1.14.6 (https://github.com/tseemann/prokka), and the resultant gff files were used for pangenome analysis with Roary version 3.13.0 (http://sanger-pathogens.github.io/Roary/) to produce gene presence and absence data. Thereafter, genes associated with host, country, ST, or clonal complex were identified with Scoary version 1.6.16 (https://github.com/AdmiralenOla/Scoary). Categorical traits were dichotomized prior to pangenome-wide association analyses with Scoary.

### Core-genome SNP analyses.

In addition to pangenome-wide association analyses, core genome SNP analyses were also performed. Core genome alignment and SNPs were detected for all 86 genome sequences using parsnp version 1.7.2 (https://github.com/marbl/parsnp). Meanwhile, exact numbers of SNPs among genomes from various hosts in the same MLST group and closely related in the core gene-based phylogenetic tree were determined with snp-dists version 0.8.2 (http://sanger-pathogens.github.io/snp-sites/) using the sequence alignment file from parsnp. The phylogenetic tree based on core genome SNPs was annotated with iTOL.

### Associations between co-occurrences of virulence genes.

Co-occurrence of virulence genes was determined with a phi coefficient using the Phi function in psych package version 2.2.5 (https://cran.r-project.org/web/packages/psych/) with R version 4.1.3 (https://www.r-project.org/); a *P* value of <0.05 was considered significant in a 2-tailed test. Pairwise phi coefficients between the presences of virulence genes were visualized using ggplot2 version 3.3.6 (https://cran.r-project.org/web/packages/ggplot2/).

### Data availability.

All whole-genome sequence data used in this study are available without restriction from NCBI under BioProject no. PRJNA848370.
