# Supplementary material for: Comparative Genomic Analyses of Lactococcus garvieae Isolated from Bovine Mastitis in China
Source: Microbiol Spectr. 2023 May 8;11(3):e02995-22. doi: 10.1128/spectrum.02995-22 (PMC10269658; doi:10.1128/spectrum.02995-22)
Supplement: Supplemental file 4 — Supplemental material. Download spectrum.02995-22-s0004.pdf, PDF file, 0.3 MB [file spectrum.02995-22-s0004.pdf]

Supplemental files

Supplemental legends

Supplemental Fig. S1 Phylogenetic tree in 86 *Lactococcus garvieae* isolates. Based on 16S rRNA with source of host (4 hosts, the first ring indicated by a rectangle) and country (16 countries, the second ring indicated by a circle) as well as sequence types (STs, 32 STs, the outermost ring indicated by a triangle) of 86 *Lactococcus garvieae* isolates.

Supplemental Fig. S2 Phylogenetic tree in 86 *Lactococcus garvieae* isolates. Based on core genes with source of host (4 hosts, the innermost ring indicated by a rectangle) and country (16 countries, the second ring indicated by a circle) as well as sequence types (STs, 32 STs, the outermost ring indicated by a triangle) of 86 *Lactococcus garvieae* isolates.

Supplemental Table S1 Forty-seven high-quality *L. garvieae* assemblies from NCBI included in our study.

Supplemental Table S2 Putative virulence genes blast results of 86 *L. garvieae* isolates.

Supplemental Table S3 The number of *Lactococcus garvieae* virulence genes.

Supplemental Text S1 The integrated *Lactococcus garvieae* virulence gene database from sequence fasta.

Supplemental Fig. S1

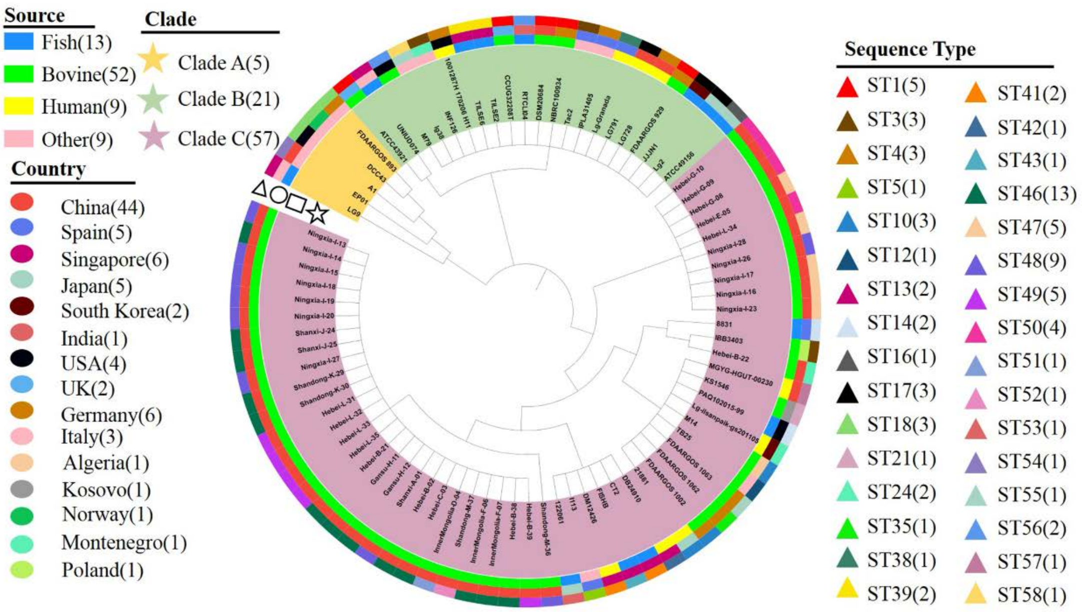

Supplemental Fig. S2

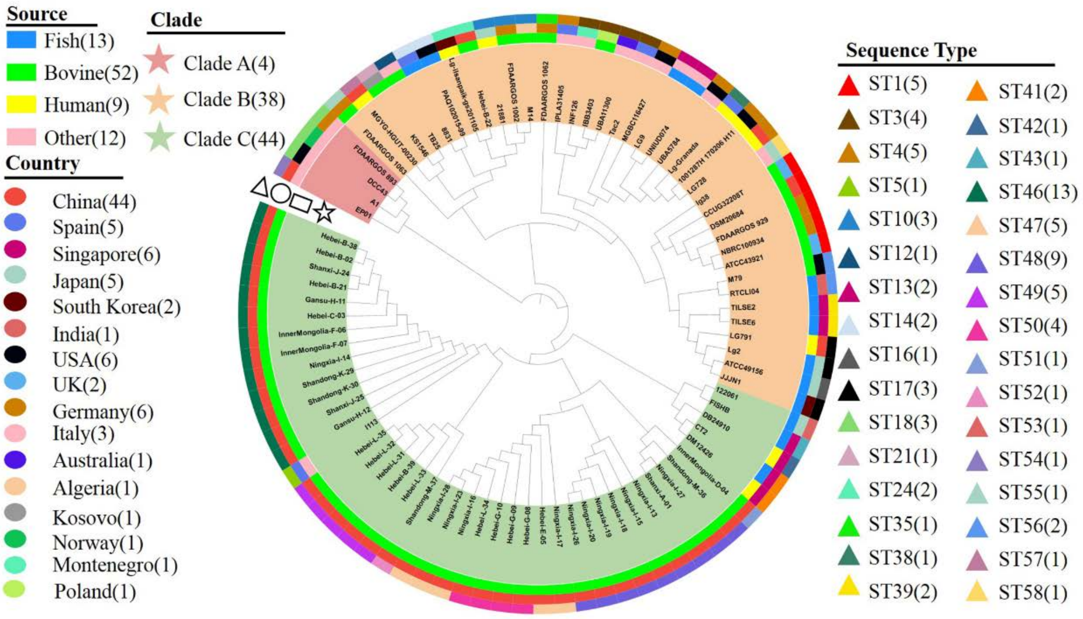

Supplemental Text S1 The integrated *Lactococcus garvieae* virulence gene

database from sequence fasta.

>hemolysin1 LCGL_RS11635F NC_017490.1:c332295-331642 Lactococcus garvieae Lg2, complete genome old_locus_tag=LCGL_0323

ATGGAAAAGCCAGCTTCTCGTGCTTATCAAATTGTCGACCAGGTTTTTAATGCCATCACACATGGCATCG
GAACCGGTCTTGCGATTACAGGACTTGTTTTACTCATACTTAAAGGGGTAGCCAATCATTCAGCGATACA
GGTTGTTGCCTTCTCAATTTATGGCGCCTCTCTTGTGCTACTTTTTCTCTTTTCAACTTTGGCACACAGC
TTACACTTCACACGCGCTCAAAAAGTTTTTCAAGTCTTTGATCATAGCGGTATCTTTTTACTCATCGCAG
GAACTTATACTCCTTATTGCCTCGTAACCCTAGGAAATTGGCTAGGCTGGGGCATGTTAGCACTTGTTTG
GCTTTGTGCAATCTTGGGTATCGTCGTTACAGCTATCTACCTGCCACGATGGAACACCGTGCCTAGAGGT
TCAACTGTCCTGTATATCGTCATGGGATGGGTCATTCTCTTTGCCATTTACCCCCTCTGGCAGCTGCTTC
CTGCCTACGGCTTTTGGTTCCTAGTCGGAGGAGGCGTGATTTACTCTGTCGGTGCAATTATTTACCGTTA
TAAATTCCCCTTCGCTCACGTGGTTTGGCATCTTTTTGTTCTTGGTGCCGCAATGTTGATGTGGTTCTCC
ATCTATGGTTATGTGGGATCATAA

>hemolysin2 LCGL_RS11890:382251-383579 Lactococcus garvieae Lg2, complete genome old_locus_tag=LCGL_0374

ATGGCACACCCTGACCCCGAGAGCACCTCGATAATTTTACAGATTTTATTACTTGTTTTT
CTTACTGCTTTGAATGCATTCTTCTCAGCAAGTGAGATGGCACTCGTTTCTCTATCACGA
TCACGTGTAGAGCAAAAAGCGAGTGAAGGCGATGAGCAGTATCAATACTTGCTTAGTGTA
ATCGATAATCCGACACACTTCCTGTCAACTGTTCAAGTGGGGATTACCTTCTTGAATATC
GTTGCGGGTGCAAGTTTGGCTGATACATTAGCTGCACAGCTGGCACCGTTGTTTGGTGAC
ACAAGCTTTGCCCAAACCTTAAGTAAAATCATTATCTTGGTTATTTTGACTTTCTTTACG
ATTGTATTTGGTGAGCTTTTCCCTAAACGTATCGCTCAGGCTCTTAAAGAAAAAGCAGCT
TTGAAAATGGTTCGTCCCTTGATGGCGATTGGTGTAGTGTTAAAACCTTTCATTTGGTTG
TTGACCATTACGATTAATGGACTTGCAAGAATCTTCCCCATCAAGTTTGACAGCAGTGAC
GATGACATGACACGTGATGAGATTGAGTATCTCGTGCACACCGATGAAACTGCCTTGGAT
GATTCTGAACGTGAAATGATTACAGGTGTTTTCAGTCTTGATGAACTTGTAGCACGTGAA
ATTATGGTTCCACGTACGGATGCTTTTATGATTGATATCAATGATGATCCACGTGAAAAT
ATTGAGAAAATGCTCAGCGAGTCTTACTCTCGTGTTCCTGTTTATGATGATGATAAAGAC
AACATCTTAGGGATTATTCACACCAAACGTATCTTAGCTCGAGGCTTTGCTGATGGTTTT
GATAACATTGATTTCCGTGAAATGTTGCAAGAACCTCTCTTTGTTCCTGAAACAGTCTTT
GTGGATGACTTGATGCTCCAGATGCGGAATACCCAAAATCAAATGGCTATTTTGCTGAAC
GAATATGGGGGCGTTGAAGGTATCGTCACACTTGAAGACTTGATTGAAGAAATCGTCGGA
GAGATTGAAGACGAAACGGATATCGCAGAAGCCGAAGTTCATAAGATTGGCGAGAACATG
TATGTGGTCCAAGGTAAAATGACCATCAACGACTTTAATGACGAATTTGGCACACATTTG
GTCAACAAAGATGTAGACACAATGGCTGGTTTCTTCCTTTCAGAAACAGGACAAATTCCA
GAAAAAGGGCAGCAAGTGATGTGTAAGATTGACAACTTAGAAGATCATTTCTCACTCACA
AGTTTAGAAGTAGATGGAAACCGTATATTAAAACTTCGTGTGGAGTTTGATGTGGAACTT
CCTGAATAA

>hemolysin3 Name=LCGL_RS12985 615478-616287 Lactococcus garvieae Lg2, complete genome old_locus_tag = LCGL_0597

ATGAAAGAAAGAGTAGATGTTTTAGCAGCAAATCAAGGATTATTTGAAACGCGAGAACAG
GCAAAACGTGGAGTTATGGCTGGTTTAGTAGTGGATGCCAAATCAGGAGAGCGTTTTGAC
AAACCTGGACAAAAGATAGAAGAAGCCACAGAACTGCGTCTCAAAGGGGAAAAACTGAAA
TATGTGAGTCGTGGAGGCCTGAAGTTAGAAAAAGCGCTTTTAGAGTTTGGTCTTTCTGTT
GAAAATAAAACGTGTTTGGATATTGGAGCTTCAACAGGTGGATTTACAGATGTCATGCTA
CAAAATGGCGCCAAACGTGTCTATGCTCTCGACGTGGGCACCAATCAATTGGCATGGAAA
CTTCGTAAAGACGAGCGTGTGGTAGTCATGGAGCAGTTTAACTTCCGCAAGGCTGTCCTA
GATGATTTTACGCAAGGTCAACCTGAGTTTACGTCCATTGATGTGAGTTTTATCTCACTT
GACTTGATTTTACCGCCTTTGTTTGATATTTTAGCTAAAGATGGCGATGTGGCTGCCTTG
ATCAAGCCTCAGTTTGAAGCCGGTCGTGAACAAGTGGGGAAAAATGGCATTATTAAAGAC
CCGAAGATCCACAAGCAAACGATTGATAAAGTGATTCAAAAAGCCTTGAGTACTGGATTC
TCAGTCAAAAATCTAACCTTCTCACCCATTAAAGGTGGTGCAGGTAATGTTGAATTTCTA
GTGCACTTAAAAAAAGAAGAGACTGCGACAGTAGCACCTCTTGTTAATATTGAAGCTGTA
CTCCAAACAGAAAAGGAAACGTTAGTATGA

>fepB putative_ferric_iron_ABC_transporter_LCGL_RS18070_NC_017490.1:c1596948-1596007
Lactococcus garvieae Lg2 LCHL_1623
ATGAAAAAGAAAACACTAACGATAATTGGTTTGCTGTTTATAGCGGTCAGTTTATCATCATGTACAAAAA
ATAAACATGTGACTCAGGAAGGAAACCAATCTCAAGTTGAAATTACGACACCACAAGGTGAAAGAGTAAA
AATTCCGCAAAATATTCAGAGAGTTGTGTCACTCTCACCCGCAGTGACACAAGTGATTAACGATCTTGGA
CAAAAAGATAAACTTATTGCTGTAGATACGCAAAGTCCTAAGTATGTTAAGGGTTTGGAAAAAGTGCAGC
AGACAGACCTTATGAACCTGGATCTTGAAAAAATAATGGCGCTCAAGCCAGAACTTTTATTTGTGAGTGA
TTTGACCCTGTTCCAATCGGAAGATAAAATTAAGAAACTCCAGGACAAAGGAACGGCAGTTGTTGTCTTA
CCTACAGAAAAGAATTTTAAAGAAATCGAAGATAATATTTGCCTTGTGGCGACAGCTTTAAACCAAAAAG
AAAAAGGGGAGCAAGTGACGCGTCAGATGGAGAAAGACATCGCTGCTTTTAAAGAACAAGCCACAAAAAT
AAGTCCTAAGAAAAGAGTGCTGTTTGAAATCGCTGCTTCTCCAGAAATTTATAGTATGGGAAAGGATACT
TATATCAATGAGATGATTGAAACGATTGGAGCTGAGAATGTGATGGCCAAAGAGGCAGGAGCAATCAAAG
TTTCAGAAGAAGCAGCTATTATGGCTAATCCAGATGTGATCTTAACAAATGTAGAATATGTCCCGGATCC
TATTAATGATATTTTAAGATTGAAAGGTTGGGAAGAGGTGACAGCAGTAAAAAATAAAGCTGTTTACTCT
ATTGATAACGAAAGAAGTTCGCTTCCCAATCAACATATAGTAAAGGCAATGACAGAAATGGCCAAAGCGG
TATATCCTGATGTATACAAAAATTTCAAATAA

>fepC putative_ferric_iron_ABC_transporter_LCGL_RS18060_NC_017490.1:c1595008-1594232
Lactococcus garvieae Lg2 LCHL_1621
ATGTTAAAAGTTGAAAATCTGAAGGTGGAGTATTCGGGAAAAGAAATTCTTTCTGATATTAGTTTTTCTG
TCAAAGCAGGGGAAAGGTTAGCAATTTTAGGGCCAAATGGTAGTGGAAAATCAACGCTTTTGAAGAGTCT
CGCTGGTTTACTAAAATATGAAGGAAAGATTGAGTTTAACGAACAATCATTGACAACATTAAAAAGAATT
GAATTAGCGGAAAAAGTGGCTCTTCTCTCTCAAAATCCAGCTGTATATTTTTCTTATTCCGTCTATGAAA
CCTTGATGATGGGGCTCTACACCCAACTGAGAAAACGATTTATGGCAGTGGCACGCGCCCAAGATAAGGC
ACGTGTAGCACAAGTGATGGTGGCGCTTGATTTGGATAAGATAAAAGATCAACAACTGTCAACACTTTCT
GGGGGCCAACTGCAGCGTGTCTTTTTTGCCCGGACATTATTACAGAACCCTCAAATTGTTTTATTGGATG
AACCCAATAATCATTTGGATATCTATTACCAACTAGAAATGTTGCGCAATATTGATGAGTATTTTACTCA
AAATCAAATCATCATTGCTGTTTTTCATGATATTAATTTAGCTCTGTCTTTTTCGGAAAATGTTTTGGTT
TTACAGGAAGGAAAAATCTTAAAACAAGGAAAAGCCAACGCGGTACTTTCTCGCTCTTTTTTGGAGCAAG

TTTACCAGACTGATGTTTTGGATTATATGCTTGAAAAACATAAATTTTGGCATGAAATTGATAAAAAGCA
AGAATAG

>fepD putative_ferric_iron_ABC_transporter_LCGL_RS18065_NC_017490.1:c1596029-1595010
Lactococcus garvieae Lg2 LCHL_1622
ATGTATACAAAAATTTCAAATAAGAATAGAATAAGCATCATTGTTCTACTTTTGGCACTGTCTTTACTGG
CAATGCTTCTCAGTCTTCGCATAGGAAGTGCTCCGATATCAGTAGGTGATTTAATGAGTATGATGATAGG
CAGAGGGAATAGTTCTGCTCCTCTTTTGCAGGACATTCTTCTTGAAGTGCGCATGCCCCGGATTTTGCTC
AGCTTTCTTGTTGGTTTAGCCTTGGGTGCGAGTGGAACTGTGATGCAGTCCCTTCTACAAAATCCTTTAG
CTTCTTCCTATACCTTAGGTGTATCATCCGGTGCTTCATTAGGGGCTTCAACTGTGATGCTATTCGGTAT
TAATTTTATGAGCCCTTCCATTTCGATGAGCCTAGCGGGCTTTGCTTTTGGACTTGTAACTGTTGTTATC
GTTTTACTCTTTGCTCAACAATTTTCCCAAGCCTTAGAGAATCAAACTATTATTTTGGTGGGAATGGTAT
TTTCTTTGTTTGTGAATTCCTTTTTAACCTTAATGATGACTTTTTCGCCGGATTATATGCAACGTATCAT
TTTCTGGCAATTAGGGAGTTTTTCGGGGGCTTCATGGGAAAACGTACAGCTCCTTGCTTTGCTTTTTACC
TTTAGTTTTTTCTGCTTGATTTACTTTCACCGAGAAATGGATATTTTGAGTTTTGGTGATGCCTTTGCTT
TGAGTCAAGGAGTAGAAGTCAAGCGTACGAAAGTTCTTTTAATTGGTTTGTCGACTTTGCTCACAGGAGC
CAGCGTAGCTATGACCGGAGTGATTGGCTTTGTCGACTTGATTGCACCTCATGTCGCTCGCCGGTTGTTT
GGGGCGACACATAAGTGGGTTCTCCCTAGTTCAGCACTTGTGGGCGGCTTGCTCTGTGTTCTTGCTGATA
CTGTGGCAAGAAGTGTTTTCCAATCAAGAGAATTACCAATTGGAGCGGTAACAGCCCTCATTGGTGCGCC
ATTCTTCTGCTATATTTTCTTTAGGAAAGGACAAGTATGA

>fecB putative_iron_transporter(iron-
heme_siderophore_like)_LCGL_RS12655_NC_017490.1:547062-548003 Lactococcus garvieae Lg2
old_locus_tag=LCGL_0530
ATGAAACACCTTAAATTATATTCTATAGCTTTAGCAGCAGTTGGACTTTTAGGCCTTTCTGCATGTTCAA
ACTCAGATGTATCACAATCCCAAAAAGAGGAAAAAGTAACCTTCACTGCCTTGAATGGTGAAGTAGAAGT
GCCTTCACATCCGGAACGTATTGCAGTACAAAATTACCCGGATGATGTTGCAACTTTAGGCGGAAATGTA
ATTGGAACGGATTCTTGGGCTTTCCCTAACCCTTATTTGTCAGATAAACAAAAGGAAAATATGGTTGATC
TTGGTTCGCCAAGCTTTAATATTGAAAAACTCATTAGTCAAAAGCCTGATTTGATTGTGACAGTGGATAA
AACACAAGTTTCTGATTATGAAAAAGTAGCGCCTACTGTACTTGTTAACTATCAAGATTTGAATAACATG
GATAAATCTTTGGATTTCTTTGCCAAATTGTTGAACCGTGAAGATGAAAAGAAAGAGTTTCTCAAAACTT
TTGACAAAAAAGCTGAAGAACAAAAAACAAAACTTAAAGCGCAAGGCATAGATACAGCACAATCAAGTAT
TTCCTTGTTAGAGCTCCAAGGCGATAAGATTTACGCTTATGGTGATAACTTTGCGCGTGGTGGTCAAGCA
CTTACGCGTGGTCTTGGATTCCAAGAATCGCCAAAAATGTCGGAATTATCCAAGGGAACAGGATATGCAG
AAGTCAACGCTGAAAGCCTCAAGGATTTTGATGCAGACTATATTTTTATTGACTTCAAAAATGCAGATAA
AGCACAATATGAGGCGCTTCAAAAAAATCCCGTGTGGAAAAACCTTAAGGCAGTGAAAGAAGGGCATGTC
ATCACCATGGATTATGACAAGGTTTATTTCTTTGGAGGTCCCACAGCTTCAATGGCGCAACTGACGGACT
ATACAGATGCACTATTAAAGCAAACGAAATAA

>fecD putative_iron_transporter_LCGL_RS12650_NC_017490.1:546112-547050 Lactococcus
garvieae Lg2 old_locus_tag=LCGL_0529
ATGAAAAAGAAATTTTTGTTTTTAATATTCCTCCTGCTTGCCTTTATTATTATCGATTTGACGCACTTTT
CGACAGATTGGGATTTGCTCTCCATATTGATTCCTCAGTTTAGAATGCCAAGAACACTAGTGGTGCTTAT

TGCGGGAACAGCTTTGGCTATGGCCGGCTTTATTATTCAGACCGTAGTGGACAATCCCTTGGCAGATGCG
GGTACACTTGGAATTACGAGTGGTGCCAGTGCAGGGGCGGTACTTTTCCTTTTTCTTAGTCAGTGGTTGA
AACTTTCAGAAACTTGGATCTTTATGTATCCTTTATTTGCCTTGCTGGGAGGACTCTTTTCCTTTGCCTT
GCTCTATCATCTTGCGCTAAAGAAAAATGTAAGTAATATCCAGGTTCTTCTGATTGGGTTAGGCATTACC
GCTCTTTTTCAGGCCCTGATTACCTTAGCTCAACTTTCAATCAATCGTTTTGATTTTCAACAAGTAGCGG
TTTGGTTATCTGGAGACATTTGGCAAACAGATAAAACGTTTATTGGTGTGGCTTTTGTTCTGCTAATCAT
TGGTCTACTTATTTTCAGTTTTTTCCGAAAAGAACTAGACTTACTAAGTTTGGGTCAAGAAATGGCAACG
AGCCTTGGATTAAATGTGAAAAAGAGTAAAATGCAATTTTATATATTGGCACTACTTTTTGCATCGATTG
GCGTTTTATTAGTTGGCGGTCTTGCCTTTATAGGCTTGATTGCACCACACATAGCTCGCGAACTGGTGGG
ATTTGAAAGTAAAAAGCGCGCCTGGGCGACTGCTTTGATCTCAATGATTATTTTACTTTTAGCAGACAGT
TTATCACAAATCATTATTGCACCGTCAAGTTTACCACTTGGTTTTGTGGTTGCTCTCATAGGGGCGCCTT
ACTATGTATACTTAATACAAAAAATTTGA

>fecC putative_iron_transporter_LCGL_RS12645NC_017490.1:545165-546115 Lactococcus garvieae Lg2 old_locus_tag=LCGL_0528
ATGAAGATTAGCAAGTTTTCTTGTCAAGTTGCACTCGCTTTGTTGGTGATTGTGGTCTTGTCTTTCCTTT
ATCTCATGCTCGGTGATCAAAACTATAGTTTCAGCCAGATTTGGCATGAGAGTGTGGTCTTGCACTTACG
TTTACCGCGCTTATTGGCTTTAGTGTTTGTAGTGGTTCTCCTCTCCAGTAGTGGACTTTTGGTTCAAAGT
ATGACAGGTAATCCTATTGCCGAGATATCCACATTGGGAATTTCAGGTGGAGCAAGTTTTGCACTTGCTC
TGCTCTTGGTATTCAATCTTTCCACAGGTGGATGGTTGGGCACTGTTGTTGCAAGCTTAGGAGCATTTAT
TGCTCTCATTACGGTAGCTGTCCTTACTGTTAAAAGTAAATTTCAACCGATGAAAGTTGTTCTTGTGGGC
ACATCTGTCGGCCTTTTTGCGACAAGCCTTGCAAGTATTTTGACCTTCTATAGTAAAAATATGCAGTCCT
ATTTTTTATGGATTGTGGGTTCATTTTCAGGGATCACTCCACTAAAACTAGAAATTTTAATGGTTGTGTC
TGTACTGTTTGTATCTATGGTTCTCTTATTTGCAAATCAGATAAAAGTCTTAGCCTTTGGTGAAGAGATG
GCAACCAGCCTGGGTATTTCTGTAAATCGTCTCAGATTGTTGATTATGGTCATGGTGGCTCTGGCAAGTG
GTGTAACTGTTTCGAGTGTGGGTGTGATTAGTTTTGTTGGACTCATGGCGCCACATTTAGCGAGACGACT
TGTTGGAGGACATTTTTTAAAAAGATTCTGTATGAGCAATTTATTGGGCGTGTTGTTATTACTTGTAGCA
GATTTATTGGCCCGTAATTTGTTCAAGCCCTATGAATTTCCGGCAGGCAGCTTAACTCTTTTATTTGGCG
CAATTTTCTTCATTTATGTGATGAGTCAGGAGGAAAAATGA

>fecE putative_iron_transporter_LCGL_RS12640_NC_017490.1:544393-545175 Lactococcus garvieae Lg2 old_locus_tag=LCGL_0527
ATGTATTTTGGGGCAAAAAATTTAAATGTATATTACGGTAAAAAACAAGTTCTAAGAGATGTATCTTTGG
AAATTGAGCAAGGAAAAACGACGGCGATTATCGGAATAAATGGCTCAGGAAAGTCGACATTACTCAAAGC
CTTGGGACGTTTAATAAAGTATGAAGGGGAAGTTATCTATAAGGAAGAACGCCTTTCTAATCGTAAAAAT
CTAGAAATAGCTAAAATTTTGACCTTGCTTCCGCAAGCCATGCAAGCCCCGAGTGATATAACTGTGTATG
AGCTGGTCAGTCTCGGCCGCTTTCCACATCAAAAACTTACACAACAGAACTTATCGGAAGCAGACAAGCA
CTTTGTGGAGCAAGTCATGAAGGAGACCGAGATTTGGGAGTTACGTCACCAAAAAGTTGCCCAACTTTCT
GGTGGCCAGCGCCAGCGGGCTTTTATCACGATGATTTTAGCGCAAGACAGTGAAATTATTCTTTTGGATG
AGCCGACAACGTATCTGGATCTTTTGCACCAACTTGATATTTTAAAACTGTTAAAAACTTTTGCGAAAAA
GATGAACAAAACAGTGGTTTATGTCATCCACGACCTCAACCACGCAGCACGTTTTGCGGATAATTTAGTT
ATTGTAAAAGAGGGGGAAATCTTCGCCTCTGGGCCGGTGGAAGAATTATTCACAGAGGAAATTATTCATG
AAAGCTTTGGATTACATGTTCGATTAGGGCGTGATCTTTTCTGCCAAACTCTTATGATTACAGGGGTTAA
AAATGAAGATTAG

>feoA putative_ferrous_iron_trasnporter__LCGL_RS10545_NC_017490.1:c109166-108705 Lactococcus garvieae Lg2
ATGAAAACACTGAATACTGCCCGTATTGGCCAAATATATTATGTAAATAAAATAATAAAGTCTGAGCACG
CGGGAAAACTTCGAGAGCTTGGGTTTGCCCCAGATAAGGAAGTCGTTTTACTTTCCATAGATGAGGAAAA
TGCGCGTTTAAAAATTGGTCAGACCCGTTTAGCGTTGAGCAGTTTCTACCTCAATACGATTTTAATTAAA
GATGAACAATCAAATGAGGAACTTGTACCTCTCTCGTCTTTGAACATCGGTCAAAGTGGTGTGGTCCGAT
TACTCGAGGGGCAAGGCGAAATAAAGCGCCGGCTTATGGATATGGGGATTACGAGGGGAACAAGTATTTC
TGTACATAAGCTTGCACCCCTAGGAGATCCTATGGAACTTCATCTGCGTGGTTATTCTCTTTCTTTAAGA
AAGAAAGATGCTGAAAAAATAAAAATTGTTGTCCAAGACTAA

>feoB putative_ferrous_iron_trasnporter_LCGL_RS10540_BNC_017490.1:c108692-106587 Lactococcus garvieae Lg2
ATGACTAAAATCGCTTTACTCGGAAACCCAAACAGTGGGAAAACAAGTATTTTCAATATATTAACCGGCA
GTAATCAGCAGGTGGGAAACTGGCCAGGTGTGACAGTCGAGTGTAAAACGGGACGTTATAAAAAAGACAA
GAACATCGCTATTCAAGATTTACCCGGAACTTACTCCCTCTCTCCTTATACTTTAGAAGAACAAGTCACA
CGAAATTATCTCAGAGAAACACCACCCGACGTAGTTCTCAATATTATAGATGCAACAAATCTCGAACGCT
CACTGTACCTCACATTGCAGCTGATGGAGTTTAATATCCCTATCGTTATCGCCCTAAATATGAGCGACTT
GCTAGAATATCAAGGTAAACAGATTGATATCGAAAAGCTATCTTACAGTTTAGGACTACCTGTTGTACTT
ACTAGTGCTTTAAAGAAGAAAGGTCTAGATGAAGCTATTCGAAAAGCTTGTCAGCCTCTTGAAGTTACTC
CTTTAGATTATGACCACCGTCTTGAAAGTGCGTTGGCAGAAATTCAAGCAATTTGTCCTCAAGCAAAGAA
TCATTTTGAACAAATCAAGCTTTTTGAAGGGGACAGTTTAGCTTTGGCCTCCCTCACTGCCTCACAAGGG
CAAGAATTAGATGAAATCGTAGCAATCACCGAAAAAATTATGGGTGACGATCGGGAATCAATCATCGTCA
ACGAGCGCTATGATTTAATTGGTCAAATTGTTCGTTTGGTTGTTACAAGCACTCATAAAGGACAACTTAA
TGTAACAGATAAAATCGACCGCATCGTTACCCACAAATGGTTGGGTCTTCCTATTTTTATCCTTATCATG
TGGCTCGTTTACTTTTTAGCGATACAGGTCATCGGTACACCCGCTAGCGATTGGCTCAATGATAACTTTT
TTGGCAGCTTTCTGCCCGATGTTTTACACCAAGCAATGGATAGCTTGGCGATTGTTCCTTGGGTCCAATC
TCTACTACTTGATGGTGTCCTCGCTGGAATTGGTGCTATCTTAGGGTTTGTTCCACAAATCTTTGTTCTT
TTCCTCCTGTTAGGCTTTCTTGAAGATTCAGGCTATATGGCTCGTGTCGCATTTGTGATGGACCGTATCT
TTAGAAGATTTGGCTTGTCAGGTAAATCCTTCATTCCCATGCTGATTTCTTCTGGCTGTGGTGTACCAGG
AATTATGGCTACCCGTACCATTGAACAAGAAAGGGACAGAAAAATTACCATCATGGTGACCACTTTCATG
CCTTGTTCAGCCAAGTTGCCGATTATTGCTTTAGTTTCAGGTGCCTTTTTTCACTCCACCAGTTGGGTTG
CACCTAGCGCTTACTTCTTAGGCATAGCCATGATTATCCTTTCAGGAATTATTTTGAAAAAAACGAGAAT
GTTTGCAGGAGATACTTCCGCTTTCATCTTGGAATTACCTACTTACCATCTGCCTCATTTTCCGACAGTC
TTTAAATATGCGTTTGATCGGGGACTGAGCTTTATTAAGCGCGCCGGGACAATTATTTTTGCGCTCAATG
TTGCACTCTGGTTCTTATCAAACTATAGTTTCACTTTGCACCAGGTCGAACCCGGCCAATCAATCTTGGC
AACGCTCGGAGGATACTTGAGCATTCTTTTTGCTCCTCTGGGCTTTGGAGAATGGAAAGCAACGGTAGCT
ACTTTGACTGGTCTAATTGCCAAAGAAACTATTGTCGGAACGATGAGCCAATTGTACGCCCAAGGTGCAG
AGGCAGAAAATAACGGCCGAGCCATTTGGACTGCTCTACAAAATAGCTACACCCCACTGGCTGCTTATTC
TTTACTTGTCTTCAACCTGTTATGCGCACCCTGTATTGCTGCGATTTCGGCAATTTATAAGGAAATGGGT
GAAGTGAAATGGACCTTACGCGCTGTGGGCTTTCAAACTGCTGTGGCTTACAGCATGAGTTTTATCATTT
ATCAGTTGGGCTCGGCCTTTCTTACGCAGTCCATTACCATCAGTACATTGCTAGCAGCACTTCTCTTGGT
TTTCGGTCTTTATCTTATCTTCCGCCGGCCAAAATATACATCGAACAAGGAGGAACTTTCTCTTGAACTT
AGCTAG

>Adhesin_PsaA NCBI_name = metal_ABC_transporter_substrate-binding_protein LCGL_RS17525 NC_017490.1:1502838-1503767 Lactococcus garvieae Lg2, complete genome old_locus_tag=LCGL_1533
ATGAACCGCAAACAGCTCTCTTTAATTGCCCTTTTAGCTTCACTTCTGTTGCTTCTTACAGCATGCTTGC
CTAAAAAAGAAGAAAGTATGGCATCTGATAAACTTAAGGTTGTAACTACCTACTCCATCATTGCTGATAT
TGCCGAAAATATCGGGAAGGACCATGTTGATGTTTATAGCATGGTGCCACGTGGCACGGATCCTCATCAG
TACGATCCGAAACCCAATGACACTCAAGCAGTTGAAAAAGCCGACCTTGTCTTTTACAATGGCCTCAATT
TGGAAACTGGAAAAGGTTGGTTTGATAAGCTGATTAAAAATAGTCGTAAAGAAGATTCAACCTTTATGGT
TAGCCAAGGTGTTACTCCCATTCACTTAAGTGAAAAAGGCAAAGAAAGTGAAGAAGACCCGCATGCTTGG
CTTAATATTCAAAACGGCATTATTTATGCCCAAAATATCGAAAAAGAACTTTCAAAGAAAGATCCTCAGC
ATAAAGAAGATTATCAAAAAAATCTCAAAGCATATACGGATAAGTTGCAACAGCTGGACACAGAAGCAAA
AGCTAAGATTGCAACCATTCTGGAGGAAGACCGAATCTTGGTTACCAGTGAAGGTGCTTTTAAGTACTTC
TCAAAACAATACGGCCTTACAGCTGAATATATCTGGGAAATCAATACAGACAACCAAGGTACACCAGCCC
AACTCAACCGTATCAACACGATTGTTAAAGATAAAAATGTAAAAGCACTCTTTGTGGAAACCAGCGTTTC
ACCAAAAACAATGGAATCTGTCTCTAGACAAACCGGTGTCAAAATCTACTCGAAAATCTTTACTGATTCA
CTTGCTGACAAAGGTCAGAAAGGTGACACCTATTACGATATGCTCAAATGGAATATTGAGCATATCACTG
ATGGGTTAAGTGGTAGGTAA

>Adhesin_Pav NCBI name = Putative_fibronectin-binding_protein_LCGL_RS16560 NC_017490.1:1326392-1328014 Lactococcus garvieae Lg2 LCGL_1330
ATGGCTTTCGACGGTATTTTTTTACATCATATGACAGCTGAAATTTCTGAAATCCTTAAAGGAGGACGCA
TTCAAAAAATTAATCAGCCTTTTGAACAAGAACTTCTTTTAACGGTTCGTTCAGGAAGAACATCACATAA
ATTATTATTATCTGCGCATCCAACTTTCGGCAGAATCCAAATCACGAAGACAGACTTCCAAAATCCTCAA
AATCCCAATAATTTTGTCATGATTTTACGCAAGTACCTGTCGGGCGCTTTTATTGAAGATATTGAGCAAG
TAGGCAATGACCGCCAATTGATTTTCCATATCTCAACCAAAGATGAAATTGGTGATGCCATGAAGATTGC
ACTGGTTGCCGAAATCATGGGGAAACACAGTAACATTATCCTTATGGATAAAAGCAGCAATAAAATCATC
GAAACCATTAAACATGTCGGGTTTTCGCAAAATCAATACCGTACTCTTCTTCCGGGATCGACTTATATCG
CCCCGCCAGCAAGCGATAAGGTCGATCCTTTTACTGCTTCTGACGAAAAGATTTTTGAGGCACTACAGAC
ACAGACCTTGCAGTCCACCTTTCAAGGTATCGGACGGGATAGTCTCCAAGCTTTAGAGAACTTGACACTT
CCAGAATTCAAGGAAAGACTGCATCACGTTCTCCCTTCTATCTATCCCAATGACAAATTTTCTGCCATTC
AACTCGCTGAAGACTATCAAGAATTTCCTAGCTTATCTGAAATGCTCGATGTTTATTATGCAGACAAAGC
TGAGCGGGATCGTGTCAAACAGGTTGCCAGCGAAGTGATCAAAAAAGTTCAAAATGAACTGAAGAAAAAT
CGCGATAAGCTAAAAAAACAGGAAGCGGAGTTACGGGCTACAGAAAATGCTGAAATCTTCCGCCAAAAAG
GAGAGTTGCTTACAACCTTCCTTCATCAAGTTCCAAACGATAAGCCTGAAGTGACTTTAGATAATTATTA
TACCAATGAGCCAATCACGATTAGCTTAAATCTTGCACTCAGTCCTTCACAAAATGCCCAACGCTACTTC
CATCGTTACCAAAAATTAAAACAGGCTGTGAAGTTCCTTGGCGATCAAATCGCCAACACTAAACAGACGA
TTTCTTACTTGGAATCTGTTGAAGCCAACCTTACACACGCCGACGTCGTTGAAATTGCTGACATCCGTGA
AGAACTCATCCAAACAGGATTTGTTAAAGCCAAATATCGTAACAATAAAAAGCAAAAAATGCTACCGCCA
GAAAAGTATCAAGCTGAAGATGGAACCATTATCCTTGTCGGTAAAAATAACCTTCAAAATGAGCAAGTCA
GCTTTAAACTCAGTCGTAAAGGTGATCTCTGGTTCCATGTGAAGGACATTCCTGGTTCACACGTCCTCAT
TACTGGCAATGCAAATCCGTCAGATGAAACGATTACTTTTGCCGGTGAACTTGCTGCTTATTTTTCAAAA

GCCCGTTATTCTAACTTGGTGCAAGTGGATATGATTGATGTGAAAAAACTTCATAAACCAACAGGAACGC
CGCCTGGTTTTGTGACTTATACAGGTCAAAAAACCATTCGTGTTACACCTGATGAGTCCGTCATCAAAAA
TGCAAGAATAAAA

>Adhesin NCBI name = Cell surface protein (putative Adhesin) LCGL_RS11015 NC_017490.1:189794-194939 Lactococcus garvieae Lg2, complete genome old_locus_tag=LCGL_0196 paper-Morita
ATGAAAAGTAAAATACCCTACAAAGTCGTACGTTTGCTTATGATTGCCTCACTTGTGGTTCAGGCGTTGC
TACCAGCATTAGTTATCGCAGAAACCATCGACAAGGAAGAAAAGCACAGTCGAACGACTTTGAGTAATGC
GCAGTGGGAAGATGAAAAGGATCTCCAGACCGTTATTGTAGAAGGAAAGGTTGAAAAGGGAACTTCCGAA
AGTAATCAACCTGAAGCTATCGTTCTGAAAGGCGCAGAATTTGAGAACGTAAGGACTGAAAAAGAACTTT
TGGGCCTCACAGAAGGTTACTATAAGTTGGAAGACAATAAGGTGCTTTTAAGCTTATCGCAGAAGAGTGA
GGGGACCTTCACTTTGAAACTTCAAGTTGTGAAAGACTCTTTAGTCAATGGAAAAGAAATCGCAGTCACT
CTTGGTGACCAAGTGTTTACTCTTCCGATTAAGTTTGAAGAAACAGACAAAGAAAAAGCTACAGCTGATG
ATAAAGCATCAGATAAGGAAGCAGAAACAAGATCTGAAGAAGGAAAAATAGAGGCTAAGAAACAAGTAGG
GAATATCACAGATAAGGAACGTGGTTTACCCTTTGCAACGACACAACCTTTGGCTAATGGTGTTCCTTGG
GACTTCGTCAGCAACAGTAAAGTGTCAGATCCTAACACAGATAGCATGGTAGACATGGGTTTCTCTGATG
TTCCCGGAGCTCATGATGGGCGTATCTGGACAGACAAAACCGTCCGTCATGATTTAGGAAGTTTAGCAGA
TGACCAGTTTGAGGTAACACTTTCAGCCTTAGCACAAAGTGCACCGATACGCGCAGGTTATCAGATCCCT
GCGGATACTGTCTTTACGATCGATGTTTCGGGAAGTATGACGGGGACGGATGGGGGAGAACGTTCACGAA
TCGCTCTTCTCGTTGATGCGCTAAATGAAGCCATCAGTATTTTACAAGAGGCTAATCCGCTCAACCGTGT
AGCCGTTGTAGCTTACGGTGGCCGTACAGGTGGTCATGCACGGGTCGAAAATATTTTGACTCTTGGACGT
TACAGTTCCAACAATGGTGCCTTCTTCATTATGGCAGGGAACACCCAAGTCAATGTTGTGGCAACGGCTG
TAGCAGGTTCAGGGGTGGCAGGTTCAAACCCTCAAGCTCAAGTACAAAACTTTGCTGTAAACGGCTCAAC
ACCCACGCAGTGGGGGATTCGCGAAGCCAGCCGTATTCTGGAAAGTGTCACTGACCAAGAAGTTGAAGTT
CCTGTGACAGATGAGAATGGAGGGGCATTACCTCCAGTACAGGTCACACGTCGTCCTAACCTCATCCTGA
TGACAGATGGTGAGCCCACCATGGGTCCGTCCAGACTTTGCCTTTGACGCTTTAACTCCAGTTGAGGTTG
GTCCAAATGGTAATCTCCTTGAAGCACCTGGTGAATTTTATGGAAATGGAAACAACGGAGAGCAAGGCCT
CGCTGTAATGACTGCCTTGACCGCAGCATACCGTGGCAAAACAGTCCTTGAACATTATTTCCCCGGTGGA
GAAGTTGGAGGTACTGCAGAGGATCAACCTGCGCCAGATGTCGGTTTCTTTAGCATTTCCTTAGGGAAAC
AAACTGAAGCTGCTCAAAACTTGATTAGCGCAACTTTGAATCCTATCCCAGATAATACAAATAAGGTTGG
TCCTAATATCTGGGCATCCACAGGTATGCCGCAACTCCCGGGTGCCCCAACTGCAGCTACACCAACCATG
ACAACCCTCTTTAATAACTTTATTGCTAATGGTTCAACAGGTAACTTCTCTGCACTCTTTAGACAATCTT
GGAACGATTATCAATGGAGAAATACGGTTAACATCAGAAATAATGCTCCTGTAACACTTGAAGCAGCAGA
TATCCCCTATGCGGATCAGTTTTTCAAGGCTGATGACCTACAGACTTTGCGCAATGCTTTTATCTCGATT
ACCAACAGTATTCAAGATACAGGCAACAGTTCAATCTTTGATGGCAATGGTGATGGGACAGACTTAGGTT
CAGGCACACTTGATTTCTCAGATGTCTTGGGCAAATACATGATCTTTGACGGTCTGACGAACATTGTGTT
CCCGGCACCAAACGGCGGAACATTTACCTATAATCTGGCAAATGTTGATGCACACCGTGACGAATTTATC
AAAACATTTACAACTCAAATCCGTCAACCTGGCACTTCCGCTAACTTTATTGATAATGCCACAGCAGGAA
CTATACTAGATAATCGTCCAGAGAATGTGATTACTTATTACTCCGATAATCAAGGCAGATATCTTGGTGT
TGATCCTGCTCTAGAAGCAGAGGCAGCTACAAAGGTACAAGTTTATCCTGTCTGGGGTGTGATACAAAAC
CAAGTAGACCCACTAGCAAATAATGTGAGTGGTCTCCTCTTTAGTGTACATACTGCGCTCAGAGCGGTTA
CTCTTGACTCAGACTATGGCTCAGTCCAACATCCTTCAGGATACAGAGAACTCGAGGCCAAGGATCAGTT
TGTACATTGGAGCATTCCTGCCAATCTTATCCCTGAACGTACTGTTCGGCAAACAGAGGATGGTATACTC

AGCATCACTGGTAACACTTCACCTATCCGTGCAAGATTTAATGTTGGTTTAGATGAAGCCAGGTTAGAAG
AAGACTTTAGAGCGGGCGTAAATGTGCCAACAAACTTCTTCTCCAACTACTGGGATAATGAGGCGAATAG
CTCTTTTGCAACATTTGAGCCGTCAAGTGAAAATCCTTTCTATCGCCTTGACGGTGCAAGAATGGTGGCG
AAAACAGAAAACCTTACAGGGACAAAACCCTATGTCAGCAAACAAATCGTAGAAGCTGATCCAACAGGCG
GACCAGATGAACGTATCGTGACCAATCTCTTGGGGAATAACGGTACATTTGCCTTGAAGTATAAAGGGCA
AATCAGACTGGAAAAAGAATTCATCTTCCTTGATGAAAATGGCGACCGAGTGAATCCTGACGGTGGTATA
CCTGCTAACGTTATACTCCGCGCATTGACCTTTACCATCACGGGTCCAAATAATTTTACACATACAGTTA
CATTTAACCCAGATGATTTCCATTTGGAAAACGGGAAATGGTACTTTGACTTGCCAGAGGACCTTCCAGC
AGGTGTATATACGATAAAAGAAGAAGGTGGAGATATTGAAACGAGTGACCCGGGTTATACACATATGCCG
GGAGGATTTGATCAAACCGTTACAGTGACGCCAGGTGGTACAGGCACAGCTAGCTTTGTCAATGTTTATG
TGCAACCTCAGCTTGCCTCACCCTCTTTACGTATCATGAAATTCTTCCATGGCTTGCCAGAGGGAACTTA
TCCGGCAGACTTTGAAATTCTTATCGAGTATCTTGGAGATGGCAACCCACAACCTGACACTGGAAATCTC
TGGACACTCAATCAAGACGGACACTACGAAACAAAAGTGAGCTTAGCAGATGCTATCGCAGGAACATCCT
TCATCGGTGTAGCCGAAGGGACTTATAGAATCAGCGAACTCAATGCGGATAATGTGAAAGACGCAGGCTA
TATCCTCTCGAACAGCGTATGGGCTTATCGCCAGCTGGGTGCTGCAGGAAATCCAGCTAACGCAGATAAC
GGACAAGGATTAGAACCGGTAGACGTGACGATTGGGGCAGCTGATGATGTGAGCTTTAGATTTGATAACT
TTTATCAAAAGTTGGGCACTTTGGATTTAACCAAAACTTTTGAAAATATCCCGCTTGATCTTATCCCAGA
AGATTTCCGTATTGTCGTGACTAACGATGCGACTGGTGAAAGAGTAGGAGAGTTAACCAAAGCAGATATT
CTTAACCAAAATACACTTCATGTAGATGCGCTGACACCAGGTTTTTATACCATCACAGAATCTAACTTTG
AGATTGAGGGTTGGGATCATATAGGTACAGCTAATATTGATGGGGCAATGCAATTTGGGACGGGGAATCC
CATCGAGTATCATTTCTCTATCCCAGGAAGCATCGGCAATAGTGATGTTGTGGTTAATATGTATAATAGC
TACGTGAACAGGCCTATTACACCACCAATTCCGCCAGTTTATCCCCTGCAGTTGACCAAGATTGACCAAT
ACGACCGCCTTGTCAATGGGGCAAAGTTTGATCTGGAAGTTTTTGATACCTCAATAGGGGATTCAGGTGA
GTGGCGAGTACTTGTTGCAGGTCTCGAGTCAGGGCAACAAACGGACGGTTTAGTCCAATATGTTGTCACA
GAACCGGGGCAATATCGTTTCCACGAAACGCAGCCAGCACCAGGTTATGAGCTAGCAGATAATCCTTACA
GTCAAGAAATAACAGTCACTGATGGTCAGGAAGGTCCGATTGACTTTGGTGAGATGGAGAACACCCTCTT
CGCACGTATTAAGTTGCATAAGACAAATGTAGAGGGCCAAGACTTATCAGACGCTATCTTCAAAATCGAA
TTCAGCGAGAGCGAAGACGGCCCGTGGGAAACTATTGTCGAAGGTCTGACAACGGAAGCCGATGGTATTG
TTGAAAAAAATGTCGGCAAAGATGGTTACTATCGTTTTATCGAAACACAAGCCCCAGATGGCTTTGTCTT
AGACAGCACCCCACGTCAAGTCCATGTCAACGCAGGAGAAACAGGGGAAACAACCTTCTTTGCTGGAGAT
ATGGTCAACCGACCAATCGCAACTCTTCAACTCCGTAAGACAGATGCGGACAGTCACGATTTAGCAGGGG
CAGTCTTTAAAGTTGAATTCAGCGAAAATAGAACTGGGCCATGGGAAACTGTCTCAAAACCAGAAGGTAT
AACTTCTGGCGAAGATGGCCTTGTTCAACTTGATGTTGAAAAAGACGGTTACTATCGTTTCATCGAAACA
CAAGCACCAGCCGGCTTTGTCTTAGACACTACGCCGCGGAAAGTCCGAGTAACCGCAGGAGTAGGCGGAC
AAGTCCTCTTCTCTGCCGGTAATATGATTAACCATAAACAACAAGGTGGAAATGAACTTAAACCACGTCT
TCCGATTGTCGGAGACAACTTGGGTCTAGGGCTCTTCGCTTGTGGTTTAATCTTCCTTCTTGCGGCAGGA
TTTATATTGTACAAGAAGAAAAAGAGTAAAATTTAA

>LPxTG-2 LCGL_RS16935_NC_017490.1:c1395289-1392494 Lactococcus garvieae Lg2 LCGT1410
ATGTCGAAATTCAAAAAGAAAAAATTACAAAAAGCTCTCACAGAAGAAAAAAATACACGTTTCCGTATGC
ATAAAAGAGGGAAGCATATGGTCACAATGTCTATGCTTGTACTCACTGTAGGTGGTTCTCTCGCAGGTCT
TGCGCAAGCAGATGTTTTAAATATCAGCCCATTTTCAAGCCCTGCACGTTCAATCTCACTTCCTGATAAA

GGGCCAGTCAACTATAAGTTTTCAGGTACTGGTACGGTCAAATGGTCGAACGGTCATGAAGCTTCCGCAA
ACTTTATGGAAGTTAATGGTGCAACAACGTTCTGTTTGGAGCCATTTGTAGATGTCTTTAATGGTGCTTA
TGCGACTAAAGCAGGTCAAAATGAAGCAGTCTATAAGTTATGGAATGAGATGACAGAGTATCAACGTAAT
CTCATTAATAACATTACTTACATTGGTGAAGTGAATAATGCAGAAGCAGATAAAAACATTAACCTTGCGA
CACAGTTTGCTATGTGGCTAGTTGAAGCAGGACAAAATGAGGTTTCAGGACTGCTTCCTAAAGTTTCAGA
AGTTGACACTTCAAAATTGAATAACGTTGTTGGTGGACATAAAATTACAGGCTTAGAGTCTACTGGTGCG
GATATTAATAAGGTCATTGAACATGCGACAGTTATTCTTAAGCAAGCAGTCGCAAGCTCTAAAAATCCAG
ATTTTAACCCTAATCCTTTGACAGTTGTTGCAGGTTCAAGTGCAACTGCCACAGATAAGGCAGGGGTTAT
TGCAGGAAATGCAGGGGGCTATGGTACCCCTTTTGATATGATACAAGCTTCAAAAGGACTTACGGCTAAA
CGAAATGGAAATTCTTTGGAAGTTTCTGCTACTCCTAGTGCGATTGGAGATAATGGTTCAGTTAAAGTTA
GAAACTACATTAATGAAGATTTTCAACCTAGCTATATTTACGGTACAATCAATCCAGACAGTACCGTAGG
ACAAACACTTTTTGCAACTTCTGACCCTGCCAATCTGAAAGGGGAATTGAAAGTTAAAACTATTGGTCTT
GGTAAGTCAACCCTTCTGAAACAAGACGCTGATACTGGCTCTGCTGAAACACAAGGTGCTGCACATCTTG
AAAACTCTGAGTGGGGGTATTTCTATAAGTCAGACGACAAACCAGTTACTTGGAAAGATGGTTATGAAGG
CTATCCTATTACTGCAGTCGCAGGTGAAAAAGTTGCAGGTGATAATGTCGTTCTTCGTATGACGGATGTT
TCTAAAGGAATTAGTGTTAAAAACTTGAAATTCCCAGATAAAGTTTACATCAAGGAAACAAAAGCACCAG
AAGGTTATGAGTTGTCAACGAAAAAATATGATGTTGAATTTGATGAAAATGACAAGTTCGATAAAGATAC
TAAAAATTATATTGAAGAAACAACTGCGACAGACCGTGTTGCTCGTTTTGGCTTTGGTTTCTCCAAAGTT
CAGGATGTCAACGGTTCTCTCACTGGCTTGAATGGTGCAGTTTTTGAAGCCAAACCTATTAATGACACAA
AGGGTGAACCTGTAGAAGTCACTTCTAGTGCAGAAACAGATTCTAATGGGGTAACGACTAACGGTATTGT
CCGCTTTAAAGACATTGTCTTTGGGGATCATCTTGTAACAGAGATAAAAACACCAGATGGCTTACAACCG
ATTAATCCCTTCACAATTACGCACACAACTAATCGTGATAAGGACGGCAACATTACAAGTTATACGTTCG
TCTTTAAAGATACAGTCACAGGTCAAATTATCTCTACAAATGATGTTGATGTGTCACACATGACAGATAA
CAATATCATGTTCAACTTGAACCTTGGTACGTTTACAGACAAGCCAGAAGAAAAACCAACACCAACCATT
ACGACTACTGCTAAGGATAAAGCAGATGGTGATAAGACACTTGGTGTAGGTCAGGCACAAGTTACAGATA
TGGCTCAAATGTCAAATCTTCCTGCTTCTAAAGAGCTGAATCTCAAAGGTAAAGTTGTTTACAAAGATTC
AGGTAAAGAAGTAAAAGATGATAAAGGTAAAGCTATTACTGCAGAAAAGAAATTTACAACGGACGAAAAC
GGTAGTGCCACAGTTCAGTTGGATTTCCCAGTCATTAACACGGTGAAAGACCAAAACAAAGACTATACCG
TCACTGAACTTGTCACAGATGAAGAAGGAAACAAGGTCGTTGAAGAAAACAACTACAAAGACAATCCTTC
TCAAACGGTTAAAGTAGATGAAGCAGATGGGCATACGGAAGTTCAGGATAAGGAAATTACACCTGAAACA
ACGACTGTAACTGACAAGTTCTTCTATGAAGGCTTGGTTAAAGGCGATACTTACACGGTTAAAATCACAC
AAGCTTATGACCATAACTTGAAAAAAGTAATTGATGTAGACGGTAGTTTGACCTTCAAAGCTGAAGATAC
TTCAGGTACTGTGGAAGTTCCTGTCAAAGTAGATGCGAAGAAATATGCAGGGCATAAGATTACCTTCTAT
GAGGACGCATGGTATGGTGAGAAACCAAATGAAGAAGAGCCACCACTGATCTCTCACCATAACAAAGATG
ATGAAAAGGAAACATTTACAGTGAAGAAACCTGAAACACCTAAAAAAGAAACACCAAAAACTCCTGACAC
TCCAAAGAGTATTCTCCCTTCAACAGGTGAAGAAAAAGCTGTTTGGAGCTTAGTAGGTATTGGTGCAATC
CTTGTAGCATTGGGAGTAGCTCGTCGTGATAAAATTAAAGCCTTCTTTAACAAAGATAAAAAATAA

>LPxTG-3 LCGL_RS17790_NC_017490.1:1548182-1549828 Lactococcus garvieae Lg2 old_locus_tag=LCGL_1585
ATGCTCCATTCAGAACACGTATCTGCTGCAGAATTAAGCAACACCAACTTTGTCGATAGTCTCAAATTTT
CAACTACTCAGCTGGCACAAGGTCAGACGACTTCTGTCAGAGTTGAGTTTAGTAGCAAGGATAATCTTAA
GGTAAAAGCTGGCGATACGATTACTTTCACACTTCCTGCCGATTTACAAGGCATGACGGAAAACGACGGT
TCTCCTCGTAAAATTTCCTTAGGTGAATTGGGAGAGGCACTGATATATAAAGACCGTGTTATTGCTACCT

TCAATGAAAAAGTTAATCAGCTTGAACATGTCAAAGGCTATTTTAATTTTGGATTACAGGCAACAAGAAC
TAAAAATCATAACGATACAAGTATCAAAACAAACTTAAGCACAACGGCAACAGCTCAAGAAATTACAATT
CATGGTGACCCAGGAAATACAGGGGAAATAGGAACTCTTCCTTTCTTTTGGAAGAGCGGTGACATGCTTG
GCGAAAAAGGAAAAGTACGCTGGTTTGTTAATGCTAATATGACAAAGGAAGAACTCTCAAGTGATATCAT
ATTAACAGATACGCACGGACTTGGGCAAAAACTTGATGCACCATCATTTCGCGTGAGCATTGAAAATTAT
TTAGGAAATTTCCAAATAACTGGCGATGAGTTTGTAGCCAAAGGATACGGCAGCATAGAAATTCTTCCCG
ATGATTCATTTATCATTACTATTAAACGTGAACATGCGCGCCTAGCCTCTTTTAGCTTCCTGTACAACAC
AATCATCACTGACAATACGGTCAAATCCTTTACCAACACATGCAATGTGAATTACCAACCCTATGGTCAA
GAAACGGTTCAAGATCAAGGCACTTCCGATGTTATAAACCTTTTCGCAGATGGTGATGCTAATGGTGAAC
AAGGCCTCAAGGAAGCCACTGAAGAAACACTCGAAAACAACTTTGAAGAGCTCGAAGAAATGCCTGCTAT
TGAAACAGAAAAACCAAACTCTTCCGTAACAGAAAATGAAGCCAATCAAACGGCGGAAAAACATCAAGTC
GACATACATGTTGCCCCTAACCAGGAAGTTGTGGAAGACGAATCAGAGCAGCTCGAAGAAATACCTGCTG
TTGAAACAGAAAAGCCAAACTCTTCTGTAACAGAAAACGAGGCCAACCAAACGGCGGAAAAACATCAAGT
CGAAAGCCATGTTGCACCTAACCAGGAAGTTGTGGAAGACGAATCAGAGCAGCTCGAAGAAATACCTGCT
GTTGAAACAGAAAAGCCAAACTCTTCTGTAACAGAAAATGAGGCCAATCAAACGGCGGAAAAACATCAAG
TCGACATACATGTTGCCCCTAACCAGGAAGTCATTACTGATGAATCTAAAAAAGTTAAAGATGTTCAACA
TCCTCAGTTTGAAAAAGCAAAACCCGTGGTGTCAGAGAAAAAGAAAAGCGAGAAGGCAGACGTACAAAC
A
CTGGGAACAAAAACAAATACTTCAAAAAGTTCTACTGTCACTCATTCAGAAAAAGTAGAAAAAAAATCGG
GTAATACTTTACCAAAAACAGGCGAGGACAAGGGGCTTATCTCTGTCTTTACTGGATTCTTTCTCTCATT
TGTTGCTTTTTCACTACGACGAAATAGACAAGCATAA

>LPxTG-4 LCGL_RS18320_NC_017490.1:c1648641-1646872 Lactococcus garvieae Lg2 old_locus_tag=LCGL_1672
ATGAAAAACTACATTAAAAATCAAGAAAAGCGTATTTCATTTTATAGCTTATTTAGAGGTATGAAGAAAA
TCCGTATAGCGGTATTACTTTTCATTAGTGCAGTGTTGTTATTCCCAAGTATTATTGCACAAGCTCAGGC
TTCTGCTTATGTAAATGCGAATACATCTTATGCGCAATCAACATATCAGCAAGAACCAATTGATGATTCC
GGTAACAGACAACTTGTCTGGAGTCTTAGAGTAAATAAAGATAATCCTGAAAATATTAATGTTAAAACAA
CAACACTCACTGAAGATTCTTTTACAGTCAATGACTATATTGATGGCTTTATATTAACAAACTACGGGAA
ACTTGCTTCAGTACCTACTGTAAGCCTCTCATCTTCAAACTTGTATAAAATACAGTACTCTATTGATGGA
GTAACCTTTACTGACAGTGCTCCAGCAAATTTAGAGTTAGTAAAAGCATTTAATGTATTAGTGGAGGGTA
CAATTCCTAAAAAAACCTCTATTGTAACAACTTTTTCTGTAAAACCAATTATGGAAGCAATTCCAGATAA
TAAGGGTGTTGTTGCTGTTAAGCATACATATAGATACGGCAGTAGTACATACGAATGGAGCCCCTTGTAT
TTTAGTCCGTTTACTGACAAAGCCGCAGACCTTACAGTAATTTATCAGGACGAGAATGGTAAAGAATTAC
ATGCGCCACAAACAATTTCCGGCAAACTTGGAGAAGCTTATGATACTACTACCTCTCAATATCAACCTTC
TATTGATGGTTATACATTAGATGAAAGTAAGCTTCCTGCTAATGCCAAAGGTACTTTATCTGACCAATCT
CAAACGGTAACTTATGTGTATACTAAGAATCCAGTCAAAGCCGCAGATGTTGCGGTAAATTATGTGGATG
AAGAGGGAAAAGCAATTCCTAGTGTTTCTCCGCAAACTATCAGTGGTAATGTAGGTGATTCCTATGATGC
TACTACGGATGTATATAAGTTAACAATTGATGGCTATACTTTAGACGAAAGCAAACTTCCTACTAATGCC
AAAGGTACTTTATCTGATCAAGTACAAATGGTAACTTATGTGTATACTAAGAATTCAGTCAAAGCAGCAG
ATGTTACGGTAAATTATGTAGATGAAGATGGAAACGCAATTCCTAGTGTTTCTCCGCAAACTATCAGTGG
TAATGTAGGAGATTCCTATGATGCTACTACGGACGTATATAAGTTAACAATTGGTGGTTATACTTTAGAC
GAAAGCAAGCTTCCTACTAATGCCAAAGGTACTTTATCTGATAAAGCTCAAAAGGTAACTTATGTGTATA
CTAAGAATGAAATACCTAATATTACTGGGATAGTTCTTGCTAAATATGTAGACATAGATGGAAACAAAAT

CTCAGAAGATATTGTGAAATCAGGAACTGTTGGAGAAGGCTATAGTACTGAAAAGAAAAAAATTAAAGGT
TACACTTTCAAAGAGGTTCAAGGTAATATAACTGGTCAATTCACAGAACAAATTCAAACCGTAACTTACG
TGTATACTAAAAATAAAGTGAATCCGGTGCTCCCTGAACCAAAACCTGAGAAGAAATCAGATTCTAAGGA
TAAAAACAATAATAAAGGAATAAAATCTTCAGACCAACACGGCTTACCTGCGACAGGGGAGAATGAAAGA
ATAACGATGATGAGCATCACTTTGGGACTAATCCTTGTAGCTTTGGCTACAGTCGTTTCTATCTTCCGCT
TTAAGAAAGCAAATAAATAA

>LPxTG-6 LPxTG_surface_protein_LCGL_RS14195_NC_017490.1:870285-870599 Lactococcus garvieae Lg2 old_locus_tag= LCGL_0844
ATGAATAATGGCAAACTAAAGGTTAGTTTAGCACTCTTAGGTTTCTCTGGTCTGTTGAATCTTTTCCTTA
CGCAACCAGTGCGCGCTAATATGATGCAAAGTAAAATTGGTTTTACAGTTCATCAAGATGTGAGAGATGC
AGGCGTATTACCACCTGAAATCCCTCAAAATGGACAGTTGGTGCATTATGATCTCTTGCCGTCTACAGGA
AGTGCTCAAGAATATTCTCTCATCTTGTTGGGCTGTATGCTGTTATTATTTGTTGTTGTATTTTTCTTAA
CTCGTATCTCTGTAGAGAAAGGAAAACTTCAATGA

>LPXTG-7 LPxTG_serin_rich_protein(putative_mucus_adhesin) _HM852546.1 Lactococcus garvieae clone SSHI.2
ACCAATGATCCGACTTTCCGTGTATGTCACGGTATCATTAACATCTGCTGAATCGGCATTCATGTCAGTT
ACGGCAGCTGTCTGCGCTTTATCAGCTACAGATCCTGGCACCACTGGGCTAACAACGGCATCAAAGGTCG
TATCACCATTTGTCGCTGTCCAGGCTGTTGTCCCAGCACCACTTGTAATAACTTCACCAGTAACGTTATC
GACAACGACTGTTCGTGTGAAGTTGACAGAATCAGTGTGTTGCTTGGCCACTACCTGTCCATTCTGATCA
ACATAGCTAATCGTCTCGTTGACCGTCTTATCATAGTTGGTGCGAGTTGGCCACTTTGGTCCATCTGGTT
CGTCTGGATTGATTGGCGTTCCAGGTGTCTTTGGATCCGTTGGATTCACTGGCGTCAACCGATGCTTCAA
GTGAACCGTGAAATTCTGATCAGTTGTGTCATCATTGTCGAACGTCAAATCTGCTGGATAGCCATCGGTG
ACCAACTCATACCCGTGTTTCTTATAATCGGCAATGCTACCGCTGGT

>LPxTG-1 LCGL_RS14985_NC_017490.1:1023654-1025762 Lactococcus garvieae Lg2 old_locus_tag= LCGL_1005
ATGAAAAAAATGACTCAGGCCATTGCTTTAAGCACGATAGTATTGGGAGCTCTCTCACCAACATTACAAG
TTATTGCTGAAACTCCGAAAATCGCAGAATCACCAGCAGTAACCACAGAGCTTTCATCAGAAAATGTTGA
AGTCCAAGCGGAATCTAATGACATCGCATCAGGAACTTTTGGGACAGCACCATGGAGAATTGATTCATCA
GGTGTGTTACATATTGGAGCGGGAACATTTTCTGACACCCCGGTTAACCAAAACGGAGCAAGACTGAGTC
CTTGGTATCAATGGAGCAAACAGATAACAAGTATCCGTTTTGAAGGTGATGTTATTGCTGGAAATATACT
CAGTAATTTATTTGATTATTTAGCAAATGTCACTCATATTGAAGGTATTGAAAAGCTTAATACTTCTCAT
ACCACTGAGATGCAACGGGTGTTTGGCGGTTGTGCGAGCTTGACGAGTCTTGACTTGACTTCTTGGGATG
TATCGAATGTTACAACAATTTTTTCTTTTCTTAATGGGGCGACAAATATGGAATCGTTAAACGTTTCTAA
TTGGAATGTTTCGAATATGATTGCAATTACTTATGCTTTTTCGGAAATGCCCAAATTAAAGGAACTCGAC
TTGTCTCAATGGCGATTAACCCCTTTATATTCTGCCCAAGGTGTCTTTATGGGAGATTCTTCTTTAGAAG
CTCTTGATCTTTCAGGTTTTGATATGACACAACTTGAAAAATCATGGATAACAGGTTATGAGATGTCGCG
TTTCTTCCAAAATACAACTTCTTTAAAGGTGCTAAAACTTAGCGATAAATTCCGTTTTTGGGTTACGGAT
AGTGTGAACGTGGAGCTTCCAGAAATTTCAGCAAACAATCAATATACAGGGAAGTGGCAAAATGTTGGTA
CAGGTTCGCTTACGCATCCGCAAGGAGCTGATGTTTGGACAAGTAAGCAACTCACTCAAAGCTTCAATGC
GAATGCGAAGAACGATACCTATGTATGGCAGCCTTACACGATATCCGCAGGCGATGTGACTGTGAAGTAC
GTCGATACAAAAGGCACGCCTATTGCAGATGATGTCATAAAATCAGGAAATATTGGTGAAGCTTATACAA

CAGAACAAAAAGATATTCCAGGCTATACTTTTAAGGAAGTTCAAGGTGATCCATCCGGTAAATTTACAGA
TCAACCTCAAACAGTGACGTATGTTTATGAAGAGGCTAAAGATCAATCAACAGTTATTGTACACGATTCA
GAGTTAACAGTTGGTGAGGCATGGCAGCCAGAAGATAACTTTGACAGTGCTACAGATTATTATGGCAATA
CGGTTCCTTTTTCAGATATAAGTGTTGAAGGGCAAGTCGATACGGCAAAAGCAGGAACTTACAAAGTCAC
GTATACAAGATTTGTACCTAATTTATTTTCAAACTCTGAAAATCAAGGGACATATTCTGCTGTTGCTACA
GTTACTGTAAAAGAAGCTCAACCGGTAAAAGGTGGGGATATTACTGCCAAATACGTGGACACAGAGGGTC
TTGAAATTTCTGATGATGTGGTAAAAGTAGGAAATATTGGTGAAGCGTATACAACAGAGCAAAAAGATAT
TCCGGGTTATACATTTAAAGAGGTTCAAGGTAATGCATCAGGTAAGTTCACTGCTCAAGCTCAAACTGTA
ACTTATGTTTATACGAAAAATGAACTCCCCCATGTGAGTGGAACAGTCCTTGTCAAATATATAGATACGA
ATGGCCATTCACTTTCGGAAGATATTGTAAAATCAGGAGCTATTGGAGAAGGTTATAGCACTGAAAAGAA
AGATATTAAGGGCTACACTTTCAAAGAAGTGCGTGGTAATGTTGCAGGTCGTTATACAAACCAAGTACAG
ACGGTCACTTATGTCTATACAAAAAATAAGTCGATTTCTCAAGTAACTGCAACTCCTCAAGGTGAAGAAA
AAGATCCAAGCGCTTCAGCAAAGCAAAAATTTTTGCCTAAGGCAGGCGAAAATGAAGGCATGACTCTACT
TAGTTTTATTTTAGGACTTACTTTCTTGGCCTTAGCCCTGTTTGGACTTACACTTCGTATTAAGAAAATT
AAGAAATAA

>Putative_collagenase NCBI name = U32 family peptidase LCGL_RS18575_NC_017490.1:1699976-1701265 Lactococcus garvieae Lg2 old_locus_tag=LCGL_1724
TTAAGCACGCACTGTTTTAGCCGCCCCTGATTTTTCGTAAAGATTGATTAAGCCTGCACCTGATTTACGA
ATCATATCTCCAGTTTGTACAGGAGTTTCCACATCAATATGTAAAAGTGTCATTGGATTTGGAGCACGAT
CGATGGATTCTCCTGTGTCTGCCAAGCGTAAGTTTTTAATTGTTGTTTCGGAATGACGGAAACCTGGCCC
ATAAAATTCAATAGTGTCGCCTTCTGTGATGACATTACGTTGACGAATTGTCGCCACCTTACTGTCCTCA
TCGTAAGCAACAACCTCGCCAATAAACTTGTATTCTGGAATTTTACGACGTGCACCAAAAAGTTGTTTAT
TTTCATCAGGAATACCATAATAAAAACCTGTATCCAACTCACGTTGCGCAACTTTCCAAAGTTCATCGAC
CAAATCTGGCTTAATAGCCTCAAATTTTTCCGGACTTTCTAAATAAGCATCAATGGCCGCTTTGTAAACA
TTTGAAACTGTCGAAACGTAGTGAATAGATTTCATACGTCCCTCAATTTTCAAGGAATTTACACCATTTT
TAATCATATCAGGAATGTGTTCAATCATGCTCATGTCCACAGCAGACATTGAAAATTCTTCGGGAATTTC
ACCTTTGATTGATTTGCGCTCACTACCAAAAGGCATATCATAAAGGTCATATTTCCAACGACACGATTGT
GAACAACCGCCGCGATTGGCATCACGCATACTCATATAGTTTGAGAGAACGCAGCGTCCACTATAAGAAA
TACACATGGCACCATGGACAAAGGCTTCAATTTCTACAGATGTATGTTTACGGATTTCCGCCAATTCTTC
CATTGAAACTTCACGGGCTAAAACGACACGTTCCAGACCTAAACCTTGCCAGAATTCTAAAGTTTCGTAA
TTCGTTGCTGAAGATTGTGTAGAAAGATGAATAGGTAAACCTGGCGCTTCGGCTGCACAAATTGCAATCA
ATGCAGGGTCCGAAACGATAACTGCAGAAATTCCTAAATCACGCAGTGTACGGAACCATTCGCCCGCACC
TTCTTCATTTCCTTCGTGTGTGACCATATTGGCCGCAACATATACCTTTGCATTATGAGCTGAGGCGTAC
TCTACGCCTTCACGCATCTCATCAAAGGTAAAGTTACCTGCGCGTGACCGTAAGCCATAGGCTTGTCCAC
CAATATATACAGCATCTGCGCCATAATCAATCGCAACTTTGAGTTTTTCCAGTGTGCCTGCAGGTGAAAG
TACCTCAGGTCTTGTATAATTTTCTTGCAT

>Sortase_A LCGL_RS16475_NC_017490.1:c1306046-1305315 Lactococcus garvieae Lg2
GTGGCGAAAAAAAAATAAAAGTTGGAAACGTATGTTGGTCAATTTGCTTATTTTGGTTCTCTTTCTTGTAG

GTTTGGTCCTTGTTTTAACAAACCCATCCGAAACTGGTTAATCGGTAGAAATACAGCGCATTATCAGAT
AGATAATGTGACACGGGAAACGATTAAAGAAAACAAAGAAGTAAAGGGCAACTTTGATTTTGACAGTGTA
GAGTCGATTAGTTTTGAGCAAGTGTTGCGCCATCAGTTAAATCGTCAACCCATGCCAGTAATTGGGGGGA
TTGCAATTCCTGATGTTGGTATCAACCTGCCAATTTTCAAAGGCTTAGCAAATGAGAATCTTGCTTTTGG
TGCTGGAACGATGAAGGAAGATCAAGTGATGGGGCAAGGAAACTATGCCTTAGCCAGTCACAATGTCACT
GGATTTAGCAGTGATGTTAGCCTACTCTTTACACCGCTTGAGCGTGCGAAAGAGGGTATGGTTATTTATG
TCACGGACAAAAATAATATCTATCAATATCGTATCAACAAAATATCTGTTGTTTCTCCAGAACATTCAGA
GGTAATCAATGATACCCCAGGAAAAACGGAGATTACCTTGGTAACTTGCGCGGATCCAGAGGCTGTCAAT
CGAATCATTGTGCATGGTACATTTGAGAAAAAAGTTGCTTATGATGCTGCGACACCAGAGATGAAATCTG
CTTTTGACCGCAGTTATAATCAAGTGCTTTAA

>Phosphoglucomutase LCGL_RS17840_NC_017490.1:1558243-1559955 Lactococcus garvieae Lg2 ld_locus_tag=LCGL_1596
ATGTCATACAATGAAATTTACGAACAATGGCTCAATGCTGACTTGACACCGGACTTGCATAAAGAACTGC
TTGCAATGGACGAAAAAACAAAGGAAGATGCATTCTATACCTATCTTGAGTTCGGTACAGCTGGTCAACG
TGGCCTTTTAGGTGCTGGAACAAACCGTATGAATATTTACACCGTACGTTTAACAACCGAAGGGCTTGCA
CGTCTGATGGACAGCAAAGGCGATGCGAAAAAACGTGGTGTCGCGATTGCTTATGATAGCCGTCACTTTT
CTAAAGAATTCGCTATGGAAACTGCAACGATTCTGGCAGACCATGGAATTCCAAGCTATGTCTATGACAG
TCTCCGTCCTACTCCCGCATTGAGCTTTACAATTCGTGAGTTGAAGACTCTTACAGGTGTTATGATTACA
GCTAGCCACAATCCTGCACCTTATAACGGCTACAAAGTTTACGGCGAAGACGGTGGACAAATGCCACCTG
AAGATGCTGCAGCCTTGACTGAATATATTCGCCAAATTGACGACATTTTCTCTATTACTTTAGGCGATAC
TGAAAAATATATCGCAGAAGGTATGATCAAAATTATTGGTGAAGATATTGATGCAAAATATCTTAAAAAT
ATCGAAACCGTTACAATCAATCATGATTTAATTAACAAATACGGTCGTGACCTTAACATCGTTTACACAC
CTCTCCACGGGACTGGTGAAATGTTGGGACGTCGTGCTCTTGCCACAGCTGGTTTTGAAAAAATTGCTGT
TGTAGAAGAGCAAGCCATTCCCGATCCAGACTTTTCAACTGTAAAATCACCGAACCCTGAAAGTCAAGCC
GCCTTTGCTATGTCTGAAGAACTTGGCCGTAAAGTTGGAGCTGATATGTTAGTTGCTACGGACCCTGATG
CCGACCGTATCGGTGTTGAAGTCCGCTTACCAGATGGAAGCTACCAACCGTTGACAGGTAACCAAATCGG
TGCCGTTTTAGCTAAATATATTCTAGAAGCACACAAAACGGCGGGCACATTGCCGGCTAATGCTGCTATG
GCGAAATCTATCGTTTCTACTGAGTTAGTCAGTCATATTGCTGAAAGCTACAACGTGGAAATGTTTAATG
TACTCACTGGTTTCAAATTCATCGGCGAAAAAATTCATGCTTGGGAAACGACAGGTGAGCACACTTACAT
GTTTGGTTTTGAGGAAAGCTTTGGCTATCTCATCAAGCCTTTCGTCCGTGATAAAGATGCTATCCAAGCA
ATGCTCCTTATCTGTGAAGTTGCGGCTTACTACCGTTCTTTAGGTAAAACACTCTATGACGGTATTCAAG
ATATCTATGCTGAATACGGCTTCTTTGTTGAAAAGACACTATCCGTTACGCTTGAAGGAAGTACCGGTAA
AGAACAAATCGCTGAAATCATGGGTAAATTCCGTGCCAATGCACCAGAAAAAATTTGACGGCCTTGAAATT
CTTTTAACTGAAGACTTCAAGGAACTTACTGCAACATCACACAGTGGTAAAGTTGAAAAATTAACCACTC
CTCCTTCTGATGTTTTGAAATATCATTTAGAAGATGGCAGCTGGATTGCTGTTCGTCCTTCAGGTACAGA
GCCTAAGATAAAATTCTATATGGCTGCTGTAGCAGATACTGAAGCACAAGCACAAAGTAAGATTGATCAT
TTTGAAAAAGAAATCACAACATTTATCGGGTAA

>Enolase LCGL_RS17440_NC_017490.1:c1490708-1489407 Lactococcus garvieae Lg2

ATGTCAATTATTACTGATGTTTATGCTCGCGAAGTCCTTGACTCACGCGGTAACCCAACAATCGAAGTTG
AAGTTTACACTGAAGACGGTGCATTCGGACGCGGTATGGTACCTTCAGGTGCTTCAACTGGTGAACACGA
AGCTGTTGAACTCCGTGACGGTGACAAATCTCGTTACCTCGGTCTTGGTACTCAAAAAGCTGTTGACAAC
GTAAACAACGTAATCGCTGAAGCTATCATCGGCTACGAAGTTACTGAACAACAAGCTATCGACCGTGCAA
TGATCGCTCTTGACGGTACAGAAAACAAAGGTAAATTGGGCGCTAACGCTATCCTTGGTGTTTCTATCGC
TGTTGCTCGTGCAGCTGCTGATGAACTCGGTGTTCCACTTTACAACTACCTTGGCGGATTCAACGCTAAA
GTATTGCCAACTCCAATGATGAACATCATCAATGGTGGTTCTCACTCAGACGCTCCAATCGCTTTCCAAG
AATTCATGATCGTACCAGTTGGTGCACCTACATTCAAAGAAGCGCTTCGTTGGGGTGCTGAAATCTTCCA
CGCATTGAAGAAAATCCTTAAAGCTCGTGGCCTTGAAACTTCAGTTGGTGACGAAGGTGGATTTGCTCCA
CGTTTCGAAGGTACTGAAGATGGTGTAGAAACTATCCTTAAAGCTATCGAAGCTGCTGGCTACGAAGCTG
GTGAAAACGGCGTTATGATCGGTTTCGACTGTGCTTCTTCAGAATTCTACGAAGACGGCGTTTACAACTA
CGCTAAATTCGAAGGTGAAGGTGGTGCTAAACGTACTGCTGCAGAACAAATCGATTACCTTGAAGAATTG
GTTAACAAATACCCAATCATCACTATCGAAGATGGTATGGACGAAAACGACTGGGATGGTTGGAAAGCCC
TCACAGAACGTTTGGGTAACAAAGTTCAACTCGTTGGTGACGACTTCTTCGTTACAAACACTTCTTACTT
GGCACGTGGTATCAAAGAAGAAGCTGCGAACGCTATCTTGATCAAAGTTAACCAAATCGGTACTTTGACT
GAAACATTTGAAGCAATCGAAATGGCTAAAGAAGCTGGTTACACAGCTATCGTTTCTCACCGTTCAGGTG
AAACTGAAGATTCAACAATCTCAGATATCGCCGTTGCTACAAATGCTGGCCAAATCAAAACTGGTTCACT
TTCACGTACAGACCGTATGGCTAAATACAACCAATTGCTTCGTATCGAAGACCAATTGGGTGAAGTTGCC
GTTTACAAAGGTCTTAACGCATTCTACAACCTTAAAAAATAA

>Glyceraldehyde-3-phosphate_dehydrogenase LCGL_RS19790_NC_017490.1:c1929013-1928003 Lactococcus garvieae Lg2
ATGGTAGTTAAAGTTGGTATTAACGGTTTCGGACGTATCGGTCGTCTTGCTTTCCGTCGTATTCAAAACG
TTGAAGGTGTTGAAGTAGTAGCAATCAACGACCTTACAGATCCAGCAATGCTTGCTCACTTGTTGAAATA
TGACACAACTCAAGGTCGTTTCGACGGTGAAGTTGAAGTTAAAGATGGTGGTTTCGAAGTTAACGGTAAA
TTTGTAAAAGTTACTGCAGAAGCTAACCCAGCTAACATCAACTGGGCTGAAGATGGTGCAGAAATCGTTC
TTGAAGCAACTGGTTTCTTCGCAACTAAAGAAAAAGCTGAACAACACTTGCACGCTAACGGTGCTAAAAA
AGTTGTTATCACAGCACCTGGTGGAAACGATGTTAAAACTATCGTTTTCAACACTAACCACGAAATCTTG
ACTGGTGAAGAAACAGTTATCTCTGGTGCATCATGTACTACTAACTGTCTCGCACCAATGGCTGATGCTT
TGAACAAAAACTTCGGTCTTAAAGTTGGTACAATGACAACTATCCACGGTTACACTGGTGACCAAATGAC
TCTTGATGGCCCACACCGTGGTGGTGACTTCCGTCGTGCACGTGCTGCAGCTGAAAACATCGTACCTAAC
TCAACAGGTGCTGCTAAAGCTATCGGTCTTGTATTGCCAGAATTGAATGGTAAACTTCAAGGTCACGCAC
AACGTGTACCAGTTCCAACTGGTTCATTGACAGAACTTGTATCTGTTCTTGATAAAGAAGTTACTGTTGA
AGAAGTAAATGCAGCTATGAAAGCAGCATCTAACGAATCTTACGGTTACAACGAAGATGAAATCGTTTCA
TCTGATATCGTAGGTATCTCTAACTCTTCACTCTTTGACGCTACTCAAACTGAAGTTACTACAGCTGACG
GCGTTCAATTGGTTAAAACAGTTGCTTGGTACGATAACGAAATGTCTTACACATCTAACCTCGTTCGTAC
ACTTGAATACTTCGCTAAAATCGCTAAATAA

>Superoxide_dismutase LCGL_RS11445_NC_017490.1:289210-289818 Lactococcus garvieae Lg2 old_locus_tag=LCGL_0285
ATGGCTTATACACTTCCAGAATTACCTTACGCATATGACGCTTTGGAACCTTTCTTTGATGAAGAAACAA
TGCACTTGCACCATGACAAACATCACCAAACATACGTAAATAATCTTAATGCAGCGATTGAAAAACACCC
AGAATTCTTTGATAAAACTGTTGAAGAATTAGTGGCTTATTTGGACCGTTTGCCAGAAGACATTCGTGTT
GCGGTACGTAACAACGGTGGAGGACACTTGAACCACACAATGTTCTGGGAATGGCTCGCTCCAAATGCAG

GTGGTGCACCAACAGGTGATATCGCTGCAGCAATCGATGAAGCTTTTGGTTCATTTGACGACTTCAAAGC
TGAATTTAAAGCAGCTGCTACAGGACGTTTCGGTTCAGGTTGGGCTTGGTTAGTTCTTGATTACGGTAAA
CTTAAGGTTGTTTCCACAGCAAACCAAGATAACCCAATTTCTGATGGCCAAATTCCAGTGCTTGGTCTTG
ACGTTTGGGAACATGCTTATTATTTGAAATATCATAATGTTCGTCCAGATTACATCGAAGCTTTCTTTAA
CTTGATTAACTGGGATAAAGTAAACGAACTTTACGCTAAAGCTAAATAA

>NADH_oxidase LCGL_RS13300_NC_017490.1:678241-679578 Lactococcus garvieae Lg2
ATGAAAATCGTTATTATAGGCACAAATCACGCAGGTATTGCTGCGGCAAATACATTATTAGACAACTATC
CTGGTCATGAAATTACCATGATCGATCGTAACAGTAACATGAGTTACTTGGGCTGTGGGACAGCCTTATG
GGTGGGCCGTCAAATTGATCAACCGAATGAACTTTTCTATGCACGACCAGATGATTTTGAAACAAAAGGT
GCGAAGGTGTTGACTGAAACTGAAGTTTCATCAATTGATTTCGAGCATAAAAAAGTGTATGCCACAACAA
AAGCCGGAGAAGAGCACGTGGAAGATTATGACAAACTCATTCTTGCGATGGGTTCAAGACCAATCATTCC
TAAAATTACAGGTAATGAATTAGAAGGTATTCATTTTCTTAAACTCTTCCAAGAAGGACAAGCAGTGGAT
GAGGAATTCGCTAAAGAAGAAGTGAAACGTATTGCCGTTATTGGGGCGGGCTACATTGGTACAGAAATCG
CTGAAGCGGCGAAACGTCGCGGAAAAGAAGTTTTGCTCTTTGATGCGGAAACTACATCTTTAGCTTCATA
TTATGATGAAGACTTTGCGAAAGGAATGGATGAAAACCTTGAAAACCATGGCGTAGAACTTCATTTTGGT
GAAACTGCCGAGGCTTTTAAAGGCACAAATGGCCGAGTTTCTCAAATTGTGACTAATAAAGGGACCTATG
ATGTGGATATGGTAATTAACTGTATCGGCTTTACGGCAAACAGCGCCCTTGTGGGAGATAAACTGGATAC
GCTTCGAAATGGAGCAGTGAAGGTAGATAAGCATCAACAGACAAGTAATCCTGATGTCTACGCAGTCGGC
GATGTGGCTACGATTTATTCTAATGCTTTGCAAGATTTTACATATATTGCTCTTGCTTCTAATGCGGTAC
GCTCAGGGATTGTTGCTGGGCATAACATTGGCGGAACAGTCCTCGAATCAGCTGGTGTTCAAGGGTCAAA
TGGTATCTCGATCTTCGGCTATAATATGACTTCTACAGGTTTTTCTGTTAAAGCCGCACAAAAATTTGGT
TTAGAAGTTGCTTACACTGATTTTGAAGATAAGCAAAAAGCTTGGTTCCTCCATGAAAATAATGATACGG
TGAAAATTCGTATTGTTTATGAAAAAATCAAGTCGACGTATCGTAGGCGCGCAAATGGCAAGCTACGGGGA
GATTATCGCGGGGAATATCAATATGTTCAGCTTGGCCATCCAAGATCAAAAAACAATTGATGAACTTGCG
CTTCTCGATTTGTTCTTCTTACCCCACTTTAACAGCCCTTATAACTATATGACTGTTGCTGCTTTAAAGG
CAGAATGA

the following are capsule gene cluster,consisting of 16 genes

>1a LCGL_RS12175 transcriptional regulator NC_017490.1:448918-449235 Lactococcus garvieae Lg2, complete genome
ATGAATGATTTATTTTACCACCGGCTAAAGGAACTAGTTGAATCAAGTGGTAAATCTGCAAATCAAATAG
AGAGGGAATTGGGTTACCCTAGAAATTCTTTGAATAATTATAAGTTAGGAGGAGAACCCTCTGGGACAAG
ATTAATAGGACTATCGGAGTATTTTAATGTGTCTCCAAAATATCTGATGGGTATAATTGATGAGCCTAAT
GACAGTTCTGCAATTAATCTTTTTAAAACTCTAACTCAAGAAGAGAAAAAAGAAATGTTTATAATTTGTC
AAAAATGGCTTTTTTTAGAATATCAAATAGAGTTATAA
>1b LCGL_RS12180 polysaccharide biosynthesis protein NC_017490.1:449269-450036 Lactococcus garvieae Lg2, complete genome
ATGATGAAAAAAGGAATTTTTGTAATTACTATAGTGATATCTATAGCATTGATAATTGGAGGTTTTTATA
GTTATAATTCTAGGATAAATAATCTTTCAAAAGCTGATAAAGGAAAAGAAGTTGTAAAAAATAGCAGTGA
AAAAAATCAGATAGACCTTACCTATAAAAAGTATTATAAAAATTTACCAAAATCAGTTCAAAATAAAATA
GATGATATTTCATCCAAAAATAAAGAAGTTACTTTAACTTGTATTTGGCAATCTGATTCAGTTATTTCTG
AACAATTTCAACAAAACTTACAAAAATATTATGGAAATAAGTTTTGGAACATCAAAAATATCACTTACAA

TGGCGAAACTAGTGAACAATTATTGGCTGAAAAAGTTGAAAACCAAGTATTAGCCACTAATCCTGATGTT
GTTTTATATGAAGCTCCACTTTTTAATGATAACCAAAATATTGAAGCAACAGCCTCACTGACTAGTAATG
AGCAACTTATAACAAATTTGGCTAGTGCAGGAGCGGAGGTAATAGTTCAACCCTCTCCACCGATTTATGG
TGGTGTTGTATACCCCGTACAAGAAGAACAATTTAAACAATCTTTATCTACAAAGTATCCCTATATAGAC
TACTGGGCTAGTTACCCAGACAAAAATTCTGATGAAATGAAGGGGCTATTTGTTGATGATGGAGTATATA
GAACATTAAATGATTCGGGGAATAAGGTTTGGCTAGATTATATTACTAAATATTTTACAGCAAACTAA
>1c LCGL_RS12185 polysaccharide biosynthesis protein NC_017490.1:450076-450855 Lactococcus garvieae Lg2, complete genome
ATGCAGGAAACACAGGAACAAACGATTGATTTAAGAGGGATTTTTAAAATTATTCGCAAAAGGTTAGGTT
TAATATTATTTAGTGCTTTAATAGTCACAATATTAGGGAGCATCTACACATTTTTTATAGCCTCCCCAGT
TTACACAGCCTCAACTCAACTTGTCGTTAAACTACCAAATTCGGATAATTCAGCAGCCTACGCTGGACAA
GTGACCGGGAATATTCAAATGGCGAACACAATTAACCAAGTTATTGTTAGTCCAGTCATTTTAGATAAAG
TTCAAAGTAATTTAAATCTATCTGATGACGCTTTCCAAAAACAAGTTACAGCAGCAAATCAAACAAATTC
ACAAGTTATTACGCTTACTGTTAAATATTCTAATCCTTACATTGCACAAAAGATTGCAGACGAGACTGCT
AAAATATTTAGTTCAGACGCACCAAAACTATTGAATGTTACTAACGTTAATATTCTATCCAAAGCAAAAG
CTCAAACAACACCAATTAGTCCTAAACCTAAATTGTATTTAGCGATATCTGTGATAGCCGGACTAGTTTT
AGGTTTAGCCATTGCTTTATTGAAGGAATTGTTTGATAACAAAATTAATAAAGAAGAAGATATTGAAGCT
CTGGGGCTCACGGTTCTTGGTGTAACAACTTATGCTCAAATGAGTGATTTTAATAAAATTGCTAATAAAA
ATGGTACGCAATCGGGAACTAAGTCAAGTTCGCCTAGCGACCATGGAGTAAATAGGACATCAAAAAGGAA
TAAAAGATAG
>1d LCGL_RS12190 tyrosine protein kinase NC_017490.1:450865-451560 Lactococcus garvieae Lg2, complete genome
ATGGCTAAAAATAAAAGAAGCATAGACAACAATCGTTATATTATTACTAGTGTCAACCCTCAATCACCCA
TTTCTGAACAATATCGTACGATTCGTACGACTATTGATTTTAAAATGGCGGATCAAGGGATTAAAAGCTT
TCTAGTAACATCTTCAGAAGCAGCTGCAGGTAAATCAACCGCAAGTGCTAATCTAGCTGTTGCTTTTGCA
CAACAAGGTAAAAAAGTACTTTTAATTGATGGCGATCTTCGTAAACCGACTGTTAACATTACTTTTAAAG
TACAAAATAGAGTAGGCTTAACTAATATTTTAATGCATCAATCTTCGATTGAAGATGCCATACAAGGGAC
AAGACTTTCTGAAAATCTTACAATAATTACCTCTGGTCCAATTCCACCCAACCCATCGGAATTATTAGCA
TCTAGTGCAATGAAGAACTTGATTGACTCTGTGTCCGATTTCTTTGATGTTGTTTTGATTGATACTCCGC
CTCTCTCTGCAGTTACTGATGCTCAAATTTTGAGTAGTTATGTAGGAGGAGCAGTTATTGTTGTACGTGC
CTATGAAACAAAAAAAGAGAGTTTAGCAAAAACAAAAAAAATGCTTGAACAAGTTAATGCAAATATTTTA
GGGGTTGTTTTGCATGGGGTAGACTCTTCTGAGTCACCATCGTATTACTACTACGGAGTAGAATAA
>1e LCGL_RS12195 tyrosine protein phosphatase NC_017490.1:451615-452379 Lactococcus garvieae Lg2, complete genome
ATGATTGATATTCATTGCCATATTTTACCGGGGATAGATGATGGGGCTAAAACTTCTGGAGATACTTTGA
CAATGCTGAAATCAGCAATTGACGAAGGGATAACAACTATCACTGCGACTCCTCATCATAATCCTCAATT
TAATAATGAATCACCGCTTATTTTGAAAAAAGTTAAGGAAGTTCAAAATATCATTGACGAACATCAATTA
CCAATTGAAGTTTTACCCGGACAAGAGGTGAGAATATATGGTGATTTATTAAAAGAATTTTCTGAAGGAA
AGTTACTGACAGCAGCAGGCACTTCAAGTTATATATTGATTGAATTTCCATCAAATCATGTGCCAGCTTA
TGCTAAAGAACTTTTTTATAATATTCAATTGGAGGGACTTCAACCTATTTTGGTCCACCCTGAGCGTAAT
AGTGGAATCATTGAGAACCCTGATATATTATTTGATTTTATTGAACAAGGAGTACTAAGTCAGATAACTG
CTTCAAGTGTTACTGGTCATTTTGGTAAAAAAATACAAAAGCTGTCATTTAAAATGATAGAAAACCATCT
TACGCATTTTGTTGCATCAGATGCGCATAATGTGACGTCACGTGCATTTAAGATGAAGGAAGCATTTGAA
ATTATTGAAGATAGTTATGGTTCTGGTGTATCACGAATGTTTCAAAATAATGCAGAGTCAGTGATTTTAA

ACGAAAGCTTTTATCAAGAAAAACCAACAAAGATCAAAACAAAGAAATTTTTAGGATTATTTTAA
>1f LCGL_RS12200 sugar transferase NC_017490.1:452401-453087 Lactococcus garvieae Lg2, complete genome
ATGGAAGTTTTTGAGGATGCCTCATCACCTGAATCGGAAGAACACAAATTAGTAGTATTAAAAAAATTTT
CTTATGGAGAGCTGATTATAAAAAGAGGGATTGATATTTTAGGGGGATTAGTGGGTTCAGGTTTATTTCT
TATTGCGGCTGCATTGCTTTATGTCCCTTACAAAATGAGCTCGGAAAAAGATCAAGGGCCAATGTTCTAT
AAACAAAAACGGTATGGAAAAAACGGTAAAATTTTTTATATTTTGAAATTTAGAACAATGATTCTTAATG
CTGAGCATTATCTAGAGCTCCATCCAGAAATTAAAGCTGCCTACCATGCCAATGGCAACAAATTAGAAAA
TGATCCACGGGTAACGAAGATTGGGTCATTTATCAGACGACACTCAATTGATGAGTTACCACAATTTATC
AATGTTCTTAAAGGGGATATGGCATTGGTTGGCCCAAGACCAATTTTACTTTTTGAAGCGAAAGAATATG
GGGAGCGCCTCTCTTACTTACTCATGTGTAAACCTGGAATTACTGGTTATTGGACAACACATGGTCGAAG
TAAAGTTTTTTTTCCTCAACGAGCAGATTTAGAACTCTATTATCTCCAGTACCATAGCACCAAGAACGAC
ATCAAGCTTCTTATGCTTACAATTGCACAAAGTATTCACGGATCGGACGCTTACTAA
>1g LCGL_RS12205 glycosyl transferase NC_017490.1:453091-454242 Lactococcus garvieae Lg2, complete genome
ATGAAGGAAAAACATATTTACATTATTGGTTCAAAAGGAATTCCAGCAAAGTATGGTGGTTTTGAGACTT
TTGTAGAAGAACTAACAGCACATCAAAGTAATAAGAACCTTAAGTATCATGTTGCTTGTTTATCAAATGA
CATACAATCAAATTTTATTCATAATGGTGCCGACTGTTTTAATATTCCAAAGAAAAATATTGGACCAGCA
AATGCCATTTATTATGATTTGGCAGCTTTAAAGTACTCACTTAAAGAAATTGAAGATAATAATTATAAGG
GTGCAATTATTTACATTTTAGCTTGCCGCATTGGTCCGTTTATTGGTCACTATAAAAAGCAAATGAAAAA
ATTAGGAATTACTTTGATGGTAAATCCTGATGGACATGAGTGGTTGCGTGCAAAATGGAGTGCACCTGTT
AAAAAATATTGGAAAATTTCAGAACAATATATGGTGAAAAATGCGGACTTATTGATCTGTGATAGTAAAA
ATATTGAGAAGTATATTCAAGAATCTTATGCGCAATATAATCCAAAAACAACTTATATCGCATATGGCGC
AGATTTAGCTCCAAGTCTTTTGAAGGATAATGACGAAAAATTAGTAAATTGGTACCAAGAAAAGGGTTTG
GAATCTAATGAATATTATCTTGTTGTAGGTCGGTTTGTTCCTGAAAATAATTATGAAATAATGATTAAGG
AATTCATGAAGTCTGATACAAAAAAAGATTTTGTTTTAATAACAAATGTAGAACAAAATAAATTTTATGA
TCAACTTAAACAGACAACTGGATTTGATAAAGATAAGCGAATAAAATTTGTGGGAACTGTATATGATAAA
GAACTACTAAAAAAAATCAGAGAAAGCGCCTTTGCATATTTTCATGGTCATGAAGTTGGAGGAACTAATC
CTAGTTTAATCGAGGCGTTAGCATCATCTAAATTAAATCTCTTACTTGACGTTGGGTTTAATAAAGAAGT
AGGAGAAAATGTAGCACTTTACTGGAATAAAGAAAATAATAATCTTGCAAAATTAATTAAACATGTTGAG
GAAACTAATTATTCTCATATGGAAAACAAAGCAAAAGAAAGAGTACAGACCTTTTTTTCTTGGGATTATA
TTATTGAAGAATATGAGAAAGTGTTTATTTAA
>1h LCGL_RS12210 glycosyl transferase NC_017490.1:454245-455555 Lactococcus garvieae Lg2, complete genome
ATGCGAATATTACATTATGGTTTAGGGTATCAACCTGAGAGAACTGGGGGATTAGTAAAGTACGCTACAG
ACTTGATGGAGGAGCAGGTAAAGCAAGAACATGAAGTCGCGTATTTATTTCCAGGAAGAATTAATATTTT
TAATTCTTCAACTAAAATAAAAAAAAGTAAAATGAGAATTACTGGTGTTACTTCATTTGAAATAATTAAT
TCCTTACCCTTAGCAGTATTTGGAGGAATTAAAAATCCAGAAAAAATTTCAATTAAAAGTTGATCATTCAA
TTTATAGTAATTTATTAGATGATTTCAGACCTGACGTGATCCATGTTCACTCATTAATGGGGATCCATAA
AGAATTTTTTGAGGTTGCTACAGATAAAGAAATAAAAATAATCTATACATCACATGACTATTTTGGGCTA
TCTCCGAATCCAACATTTTATTTTAATGGTAAGTCATGGGATGAAGAAAATGATTTAAATTTCTGGTTAA
ATGTTAGCCAGGGAGCACTCGGATTAAAAAAAATAAAAATACTTCAGATGCCATTTTATTCAAATATTCG
AGATATAATTAAAAAATTTAAAAAAAGTTCTTTTCAAAAAGAAAAACATTTCAAAACTATGGAAAACGAT
GACTTCTCTATAGATTACCAAGAATCATTTAAAGAACTGATTGATTATTATCAAGGCATATTTAGATTAG

TTTCTTTTTTCCATTTTAACAGTAATATTTCCAAAAAGGTTTTTGAGAGAAATTTAATAGAACAAAGTTT
TAATTATGAAGTACTACCAATCTCAAATTCAACTATTTTGGGTGGAAAAAAACATATAGAGTTAGATATG
TCAAAAGTAAAAAAAAATCACTTATATCGGTCAATATGCTGAATTTAAAGGACTATACGATTTCCTAAGTT
TAGCTAATTATTTTAAAGATAGTAATATAACATTTGAAATATTTGGAGAAGATATTGATGTTGAACTTCC
TCAAAATGTTTCAAACAGAAAAAGATTTTCACCAGACAATATAGAAAAGATTTTTAAAAATATCCAGTTG
TTAGTAATCCCAAGCAGATGGAAGGAGACCTTTGGTTTCTTATCATTAGAAGCTTTATCTCACGAAGTGC
CTATTATTGTTTCATCCAGTATCGGATCGAAAGATATAATACCTGATATATGTGTTTTTGAATCTCAAAG
TTTATCAAGTACTATGATGGACTGGGTTTCTAAAGATAAAAAAATTCAGTTTGTTATAGATTTAAAAGGT
ATGCAAGACCATAATAAGGAGATAATTAATTTATATGAAAGAATTATTTAA
>1i LCGL_RS12215 acetyltransferase NC_017490.1:455539-456228 Lactococcus garvieae Lg2, complete genome
ATGAAAGAATTATTTAATAAAAGTATCACAAAAATATGGGGAGGGAGTTTTGAAATATCAAATGATATAC
CATCATCTTATATTATTTCGCTTGTTTTTGAGACCCTTTTCAACGTGATAAGAGGTCTAGTGAAAACACT
TGGATTCGGTGAAAAAAGGGGGCTTGTTTTTGTTGGAAAAAATGTCACTTTAAAAATGAGACAAAAAATT
TTTGTAGGTTCTAAAGTAAGATTTGAAGAGGGGTGTGAAGTTATTGCCTTAAGTTCTGAGGGGTTTCATC
TTGGAAACAATGTTAAAATTGGTCCGCATACAAAAATGATCTCGGGATCAATTAGTAGTATTGGAAAGGG
TATTTATATAGGGGACAATTGCTTCTTTTCTGATTATACTTTTTTTGGGGGAGCAGGTGGTATAAAAATT
GGAAGTGATATAATTTCAGGCCAAAATGTTAGGTTTCATGCTGAAAACCATAATTTTTTGATAAAAGATG
AACTAATAAGAAATCAAGGAGTTAACCATAAAGGAATCGAATTAGGAAATGATATTTGGATTGGATCAGG
TGTAGTTTTTTTAGATGGCGCTAAAGTGGGGAACCATTGTGTTATAGCTGCAAATACAGTATTAAATAAA
GAATATCCAGATTCTTGTATTATAGGAGGTGTTCCTGGGAGGGTAATAAAAAAATTATGA
>1j LCGL_RS12220 glycosyl transferase NC_017490.1:456225-457340 Lactococcus garvieae Lg2, complete genome
ATGAGAAAAAAAATTATGATAGTTCGGAATGGACCATATCCTGTAGACATTAATGCATATAATGATCAAT
TTATTGGTCTAGCTAGGGCATTTGCTAAAGTAGGATATGATTCAGATATATTTTATTATTCAAAAAAAGA
TGAAAATACAATTGATATTTCAGTTGATAATGCAGCTATCAGAGTATACAACACGAGAGGGATTAGAGTA
TTAAGAACGGGAATATACCCCAAGCTATTAAGTAAAAAATTTCAGAATAAGTACGATTTGATTATAACAA
CAGAATATAGTCAAATAATGACTTTTTTGTTTTCTTTCTCACATATTCCGGTAATTCTATACACCGGTCC
ATACTATAATTTATTTAAGTTGCCTTTTCTGTCTCCAATATATGATCTACTTTTTACCAAAAGAATTGAT
AAAAAAATGAAAAAAAATCCTTACAAAATCAGAATTAACCACAGAATTTTTAACAAAAAAAGGATACCATA
ATATTACGACAGTAGGAGTGGGACAGGATGTGGATCGATTTTCTGAAAATGTAGTTATTAGCAAAAAAGC
AAAAAAGTTATCTACTTTTATGGAAAATAATAATTGTATTTTAACTGTTGGAAGCATTGATTCCAGAAAA
AATTTTCCATTTATTTTAGATGTTATGGAGAAACTAATCAAGAAAGATAATTCTTACACTTTAATATGTA
TTGGTACGGGAGATGAAAAATTTATATCTAGAGAGTTGTTAAAACATTCTGAGCAATTGAGAGAGAGAAT
TATATTTCCTGGTAAATTTGATAATAGAGAATTAAAAAACATATATCCGAAAGCAAAAATATTTATACAT
CCAGCAAAGCTAGAGATTTTTGGGATGGTATTATTGGAAGCAATGTATTTCGGAACAATTGTATTGTCTT
CACCTAATGGTGGTTCAACAACACTCATAAAGAATGGAGAAAATGGATATGTTTTAGATATTGATAATGT
TGATAATTGGGTAAAAAAAAATAGTTGAAATCGACAATAATAGAGAATTAAAAAGTAAAATTATAAGTAAT
GCAAAAAAAAACTATAGAATCCGATTATACTTGGGATAATATTGTCAAGTATTTTTTGAATAAGTAA
>1k LCGL_RS12225 hypothetical protein NC_017490.1:457405-458526 Lactococcus garvieae Lg2, complete genome
ATGAATATTAACAAAAAAAATAGCATCATATAGAGGAGGTATTATTGGAGGAGCAGTTATCCTCATTTCAC
TTAATTCAGCATTATTTATTAACTTAAGCTCATTGACAATTTATTATATTTCAAAAATTATTGTTATGGT
TTTGATAATATTAAGTCTTATTTTAAAACGGTGGACCAAAAGAAATGTTTTTCTGTTTAGTTTAGTTTTT

ACCATAGTACTATCCGTTTATAATATTACTAGAGATGATGGATTAATATTTTTATCAAGTTTTGTATTTT
CAATAGGAGAATTAAATATAAAAAAATTAGTTAGAATATCACTGATAGCAAGATTAATGGGTGTTTCAAT
AACATTTTTATTTTTTATATTGGGTTTTCTTCCTCATCTTATCTACTTAAGAGGGGCTGATCAACGTTTT
AGTTTGGGTTTTATCCAACCTAATTTACTCTTTGCAAATATATTTTATATAATATTAGGATTAGTATTTC
TGAGATTTGGTAAAATAAAAATTTATGAAATTTCTTTTTATTATATAATTTCTTTTATCTTTGCTCAGAT
CTCGGGCAATAGGACAGGATTTTATTTAATGTTTCTATTTTTGACTTATGTTTTATTTTGTTCGAATAGA
GATAAATTTTTATTTATTGTAAAAAGAAATTGGTGGGTTATTTTCTTTTTTGTATCTATTGCTTTATCGC
TAATGTATTCACCTTATAATAATTTGATTGCGTTATTGGACGCAGTAACTGCTGGACGTATTAATCAAGC
TAATTATTTTTTAAATAATTTCCCCATTACTCTCTTTGGAAATAAAGTGACAAAAATGTATACAACGGTC
TCATATACTGGTAGTGTCCATATACTTGATAATTTATATGTGAGTCTCTTAGTCCAGCAAGGTATCTTGA
TAACAATCGGGGTTTTAGTATCAATTTCAGTCATGATCAAGAAATTGAAAAATAGTATACCTAATGAAAA
AATAGATAATAATTTGATAATCATGTTATTCTTTATTTTTGGTATGTATGGTTTGTTTGAAAAATCACCG
TTGGATATTAGTTATAATATATTTTTATTATATTTTTCATTAATTTTAAAAAAAGGAAATAACTATGATT
GA
>1l LCGL_RS12230 lipopolysaccharide biosynthesis protein NC_017490.1:458519-459955 Lactococcus garvieae Lg2, complete genome
ATGATTGAACAACTAAGAAAAGGATTAATCTATACTACACTGGGGCAATATGGAAATGTACTAGTGAATT
TTATAGTAAATGCTATATTGTCTAGAATACTAACCCCGAAAGAATATGGCATAGTAGCAGTAATAAGTGT
TTTTATAGTATTCTTTCAGGTTTTGTCTGACATGGGAATTGGTCCTTCAATAATACAGAATAAAATGTTA
ACGCAAGTTGATGTAAATAACATTTTCGGATTTTCCTTTTATTTTTCTTTATTACTTTCTGTTATATTTA
TTTTTATAGGTGTGTTTCTTAGTACTGCATATTCAAACCCAATATATAATAGCTTGTCTCAAATTCTTTC
AATTAGTCTGTTTTTTTATACACTGAATATAGTGCCCCAAGCTATCTTAAAAAAGGATAAAATGTTTAAA
ACCTTAAATATTATTTTGGTGGTATCAGCACTCTTTTCAGGTTCTTTTGGTATTTTGTTTAGTTGTTTAG
GTTTTGGAGTTTATTCTTTAGTTTGGATGACAGTAATCAATTCAATTATGGCATTTATATTTACATTTAT
TAATTCAAAAATTTCGTTTAAGTTAACGTTTAGAAAAGAATCCTTAGCAATGGTAAGTGGTTTTGCAAAG
AATCAATTTGGATTTGGTATTGTAAATTACTTTTCAAGGAACACGGATACAATATTAATTGGTAAGTTTA
TTGGAGGTGAGGCTGTGGGAAGTTATAATAAGGCATATCAGTTATTAATGTATCCAGCAACTATATTTAA
TGGAATAATCAATCCTGTACTTCAACCAATATTGTCAGACTTTCAGGAAGATGTTGTGAGAATTAAGACT
ATATACTTGAAAATTATTCATTTTTTAATGTTATTGGGTATACCAATATCAATTTATCTATCCATGAATT
CACAAAGAATAATTTTCTTTATATTTGGTAATCAATGGAGTGATAGTGTTTATCCATTTTCTATTCTCGC
ATTAAGTATATGGACTCAGATGGTAGCAGTAACGACAAGTTCAATATTTCAAGCGAGAAATAAAACAAAC
TTACTATTAATTAGTGGACTAATTAATTCAACAATAATTATTACTTTAGTTATTATTGGGGTAATATTCC
AATCAATTGATTTAGTTGCGACGCTGTTGTCGTTTGGATACTTTTTTACTTTATGTGTAACTTTTTATTT
TTTACTATATAAAGTTTTAAACAGTAATTTTTATGAGTTAGTTAATGAAATGATAAATTCAATTTTTATA
GGAATGGGAACTTTTATAACTATGTTATTATATAATGAATTTACTCAAGAGAGTAACTTCGGATCTTTAG
TTTCTAGTTTTATTATTTTTAGTTTTATCTATTTTATTCTTTTATATTATACAAAAGAGTATAAATTTAT
TTTAAGTTTCATAAATAAGAAAGAGGAAAAACAATGA
>1m LCGL_RS12235 UDP-glucose 6-dehydrogenase NC_017490.1:459952-461118 Lactococcus garvieae Lg2, complete genome
ATGAAAATTGCAGTCATTGGCACAGGTTATGTCGGCCTTTCTATTTCGGTACTTCTAGCACAGCATCATG
AAGTCATTGCATTGGATATTGTTGAATCGAAAGTAGATTTGATTAATTCTAAAAAATCACCTATTGTCGA
CAAGGAAATTGAGATATTTTTAGCAAGTAAAGAATTAAATTTATTAGCTACAACGAATAAAGCGCTAGCG
CTTATGAAAAGTGATTTCGTAGTTGTAGCAACACCTACCAATTATGATGATGTCAAGAATTATTTTAATA
CGGATTCTGTAGAAGCGGTGATTGAAGAGGTTTTGGAGTTTAGTCCAAATGCTACGATTGTAATAAAATC

AACTATTCCAGTTGGTTTTGTGGAAAAAATGCGTGCTAAATATAATATTGATAACATTATCTTTTCACCA
GAATTTTTACGAGAAGGTCAAGCGTTATATGACAATCTTCATCCATCTCGCATTGTTGTTGGTGAGCAAT
CTAAGCAGGCCTCTCAATTTGCTGAGTTATTAATAGAGGGAGCTGTTGAGAAAAATATTCCAGTTCTTTT
TACAAATCCTACTGAAGCGGAGGCAATTAAGCTATTCTCAAATACATATTTGGCTTTACGCGTTGCTTAT
TTTAATGAATTAGATACCTATGCTGAAGTTCGTGGTTTAAATACAAAACAAATAATTGATGGCGTTGGGC
TAGATCCACGAATTGGAACCCACTATAATAATCCTTCATTTGGTTATGGAGGGTATTGCCTACCGAAAGA
TACCAAACAGCTTTTGGCTAACTACGACCAAGTTCCTGAAAAATTAATTGAGGCTGTTGTTGAATCAAAT
AGTACAAGAAAAGATCATATTGCAGATATGATCATTAAACGTTCTCCTAAAGTGGTGGGTATCTACCGCT
TGACTATGAAGTCAAATTCTGATAATTTTAGATCAAGTTCAATTCAAGGAATTATGAAACGAATTAAGGG
CAAGGGAATTGAAGTAATTGTCTATGAGCCAACACTAAATGATACTAATTTCTACAATTCTCGGGTAGTT
CATAATCTGGATGAGTTTAAAACGATTTCTGATGTGATTGTTTCAAATCGTTATACTAAAGAACTTGAGG
ATGTTAAAACTAAAGTTTATACGAGAGATTTATTTGGGCGTGACTAA

>1n LCGL_RS12240 exopolysaccharide biosynthesis protein NC_017490.1:461140-462042 Lactococcus garvieae Lg2, complete genome

TTGGAACGAAAAAAAAAGAAAAAAAAGAATATTTGGGTTATAATTATACCTATCTTAATTTTTATTACCT
TTATAGGAGCAGGGGCTTATGCTTTAAGAGATTCACTTATTCCTACTGATCATACGAAAACAAATAGTTC
GGATCAACCGACCAAAACTTCGTCCTCTAATGGTTATGTAGAGAAAAAAGGTGAAGAAGCTGCTGTGGGT
AGTATAGCACTTGTAGATGATGCTGGTATACCAGAATGGGTTAAAGTTCCCTCAAAGGTAAATCTAGATA
AATTTACTGATTTATCTACGAATAATATCACTATTTTTCGAATTAACAATCCGGAAGTCTTAAAAACAGT
TACCAATCGTACAGATCAACGGATGAAAATGTCAGAAGTTATAGCTAAGTATCCTAATGCTTTGATTATG
AATGCTTCCGCATTTAATATGCAGACAGGACAAGTAGCTGGATTTCAAATTAATAATGGAAAGTTGATTC
AAGACTGGAGCCCAGGTACAACGACTCAATATGCTTTTGTTATTAACAAAGATGGTTCGTGCAAAATTTA
TGATTCAAGTACACCTGCTTCAACTATTATTAAAAACGGAGGGCAACAAGCCTATGATTTTGGTACTGCA
ATTATCCGTGATGGTAAAATTCAACCAAGTGATGGCTCAGTAGATTGGAAGATCCATATTTTTATTGCGA
ATGATAAAGATAATAATCTCTATGCTATTTTGAGTGATACAAATGCAGGTTATGATAATATAATGAAATC
AGTGTCAAATTTGAAGCTCCAAAATATGTTATTACTTGATAGTGGTGGCTCAAGTCAACTATCTGTCAAT
GGTAAAACGATTGTTGCTAGTCAAGATGATCGAGCCGTACCGGATTATATTGTAATGAAATAA

>1o LCGL_RS12245 LytR family transcriptional regulator NC_017490.1:c462969-462067 Lactococcus garvieae Lg2, complete genome

ATGAATCAAAAAAAGAGGCGTCATTATCGTAAGAAAAAACACACAGTACTAAAAGTTATTTCAATTATTT
TTGTATTAGTAATTATCGCTATTGCTTCTATAGCCTACGCTGCTTATAGAAATGTTGAATCAACATTTTC
AACATCATATGAAAATTTCCCTAAAACAACAAGTATTGATATAAAAAAATCTAAAACATTCACCACACTC
ATCATTGCAACTGGTAAAAATAATTCTAAAAATACAGCTTATGCTACTGTTTTAGCTTCAACGAATGTAA
AGACAAATCAAACTACTTTCATGAACTTCCCAGTTTTTGCGACAATGCCTAATCAAAAAACAATCACTGA
AGTTTACAATACGAATGGAGATGATGGAATTTTCCAGATGGTTAAAGACCTATTGAATGTGTCCATTAAC
AAAGTAATTCAGATCGATGTTAATAAAATGGGATCACTTGTACAGGCCACTGGTGGAATCACCATGCAAA
ATCCAAAGGCATTCAATGCTGAAGGTTATGAGTTTAAACAAGGAACTGTTAATTTACAAACTGCTGATCA
AGTCCAAGCCTATATGACACAAATTGACGATACTGATTTGGATGCTTCAATCACTCGGATTCAAAATGTC
TCAATGGAACTCTACGGAAATATTCAAAAAATTGCTCATATGAAAAAACTTGAAAGTTTCAATTACTATC
GAGAAATTCTCTATGCTTTTTCAAACACTGTTAAAACCAATATAAGTTTCAATGATGCTAAAACGATCGT
TATGAGCTACAATAAGGCTCTAAAGAATACCAGCAAGCTCAATCTACATACAACAGATGAAAATGGAGCT
AAGGTCGTTTCTCAAACAGAATTAGACTCAGTCAAAACCCTTTTTGAAAAATCTCTAAAATAA

>1p LCGL_0447 truncated transposase NC_017490.1:463075-463200 Lactococcus garvieae Lg2, complete genome

ATGCAAAAACGCTACTCAAAAGAATTTAAAGAAACCCTTATCGCCTTCTATCATTCTGGTCAATCCGTCA
CCCAGCTGTCTAAAGAATACGACGTGGCCCCTGCTGGACTATTATCATATAATTAG
